# Indole-3-propionic acid promotes hepatic stellate cells inactivation

**DOI:** 10.1186/s12967-025-06266-z

**Published:** 2025-03-01

**Authors:** Mariana Ilha, Ratika Sehgal, Johanna Matilainen, Kirsi Rilla, Dorota Kaminska, Shrey Gandhi, Ville Männistö, Charlotte Ling, Stefano Romeo, Päivi Pajukanta, Eija Pirinen, Kirsi A. Virtanen, Kirsi H. Pietiläinen, Maija Vaittinen, Jussi Pihlajamäki

**Affiliations:** 1 Institute of Public Health and Clinical Nutrition, Department of Clinical Nutrition, University of Eastern Finland, Kuopio, Finland; 2 Department of Neurosurgery, University of Pennsylvania, Philadelphia, USA; 3 Department of Experimental Diabetology, German Institute of Human Nutrition Potsdam-Rehbruecke (DIfE), 14558 Nuthetal, Germany; 4 Institute of Biomedicine, School of Medicine, Faculty of Health Sciences, University of Eastern Finland, Kuopio, Finland; 5 Department of Medicine, Division of Cardiology, UCLA, Los Angeles, CA USA; 6 Institute of Immunology, University of Münster, Münster, Germany; 7 Department of Genetic Epidemiology, Institute of Human Genetics, University of Münster, Münster, Germany; 8 Departments of Medicine, University of Eastern Finland and Kuopio University Hospital, Kuopio, Finland; 9 Epigenetics and Diabetes Unit, Department of Clinical Sciences, Lund University Diabetes Centre, Scania University Hospital, Malmö, Sweden; 10 Department of Molecular and Clinical Medicine, University of Gothenburg, Göteborg, Sweden; 11 Department of Human Genetics, David Geffen School of Medicine at University of California Los Angeles (UCLA), Los Angeles, CA USA; 12 Institute for Precision Health, School of Medicine, UCLA, Los Angeles, CA USA; 13 Research Program for Clinical and Molecular Metabolism, Faculty of Medicine, University of Helsinki, Helsinki, Finland; 14 Research Unit for Biomedicine and Internal Medicine, Faculty of Medicine, University of Oulu, Oulu, Finland; 15 Medical Research Center Oulu, Oulu University Hospital and University of Oulu, Oulu, Finland; 16 Biocenter Oulu, University of Oulu, Oulu, Finland; 17 Turku PET Centre, University of Turku, Turku, Finland; 18 Obesity Research Unit, Research Program for Clinical and Molecular Metabolism, Faculty of Medicine, University of Helsinki, Helsinki, Finland; 19 Obesity Center, Endocrinology, Abdominal Center, Helsinki University Central Hospital and University of Helsinki, Helsinki, Finland; 20 Department of Medicine, Endocrinology and Clinical Nutrition, Kuopio University Hospital, Kuopio, Finland

**Keywords:** Indole-3-propionic acid, Liver fibrosis, Hepatic stellate cells, Apoptosis, Mitochondrial metabolism, Epigenetics

## Abstract

**Background & aims:**

We have previously reported that the serum levels of gut-derived tryptophan metabolite indole-3-propionic acid (IPA) are lower in individuals with liver fibrosis. Now, we explored the transcriptome and DNA methylome associated with serum IPA levels in human liver from obese individuals together with IPA effects on shifting the hepatic stellate cell (HSC) phenotype to inactivation in vitro.

**Methods:**

A total of 116 obese individuals without type 2 diabetes (T2D) (age 46.8 ± 9.3 years; BMI: 42.7 ± 5.0 kg/m^2^) from the Kuopio OBesity Surgery (KOBS) study undergoing bariatric surgery were included. Circulating IPA levels were measured using LC–MS, liver transcriptomics with total RNA-sequencing and DNA methylation with Infinium HumanMethylation450 BeadChip. Human hepatic stellate cells (LX-2) where used for in vitro experiments.

**Results:**

Serum IPA levels were associated with the expression of liver genes enriched for apoptosis, mitophagy and longevity pathways in the liver. AKT serine/threonine kinase 1 *(AKT1)* was the shared and topmost interactive gene from the liver transcript and DNA methylation profile. IPA treatment induced apoptosis, reduced mitochondrial respiration as well as modified cell morphology, and mitochondrial dynamics by modulating the expression of genes known to regulate fibrosis, apoptosis, and survival in LX-2 cells.

**Conclusion:**

In conclusion, these data support that IPA has a plausible therapeutic effect and may induce apoptosis and the HSC phenotype towards the inactivation state, extending the possibilities to suppress hepatic fibrogenesis by interfering with HSC activation and mitochondrial metabolism.

**Graphical Abstract:**

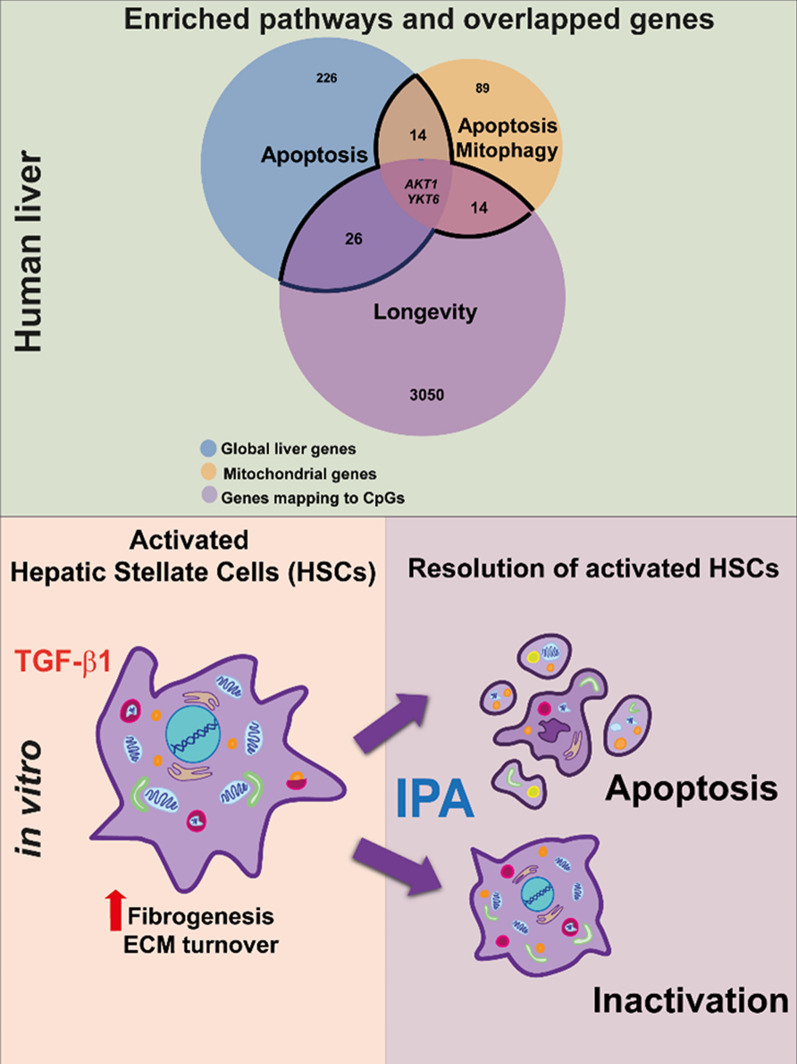

**Supplementary Information:**

The online version contains supplementary material available at 10.1186/s12967-025-06266-z.

## Introduction

The spread of obesity and metabolic syndrome is linked to increased metabolic (dysfunction) associated fatty liver disease (MASLD) incidence; affecting 25–30% of the general population [1]. A major consequence of MASLD etiology is liver fibrosis which is a dynamic process characterized by continuous accumulation of fibrillar extracellular matrix (ECM) [2]. The main cells involved in liver fibrosis are hepatic stellate cells (HSCs), having four known phenotypes: quiescent, activated, inactivated, and senescent [3, 4]. HSCs can activate and transdifferentiate from a quiescent phenotype into a high energy demand proliferative fibroblast-like shape with increased expression of α-smooth muscle actin (α-SMA) and type I collagen (Col-I) [5, 6]. During liver fibrosis reversion, activated HSCs are cleared through apoptosis or inactivation. These processes involve the downregulation of fibrogenesis genes and modulation of survival genes like NF-κB and PI3K/Akt signaling [7, 8], together with modifications in mitochondrial dynamics and function [9].

The serum levels of gut-derived tryptophan metabolite indole-3-propionic acid (IPA) are found to be reduced in metabolic diseases including MASLD in humans [10–13]. IPA is associated with dietary fiber intake and is known to induce anti-oxidative, and anti-inflammatory effects, and attenuate diet-induced nonalcoholic steatohepatitis (NASH) phenotypes in vivo and in vitro [11–14]. Part of this evidence comes from our previous study demonstrating that serum IPA levels were lower in individuals with liver fibrosis compared to those without fibrosis in obese subjects from the Kuopio OBesity Surgery (KOBS) study. Furthermore, we showed that IPA treatment was able to reduce cell adhesion, cell migration, and the classical markers gene expression for HSCs activation in the human hepatic stellate cell model (LX-2), being a potential hepatoprotective metabolite [15]. However, how IPA induces the regression of liver fibrosis through activated HSCs apoptosis and mitochondrial bioenergetics is still unknown.

Here we demonstrate that serum IPA is associated with the expression of genes enriched for apoptosis, mitophagy, and longevity pathways in the liver of individuals with obesity but without T2D (KOBS). Additionally, we found that IPA could induce the clearance and resolution of activated HSCs through the inactivation pathway. These findings reveal a novel role for IPA, rendering the compound a potential therapeutic target driving the regression of liver fibrosis.

## Materials and methods

### Study population characteristics & liver histology

Circulating IPA levels were found to be lower in individuals with liver fibrosis when compared with those without in the KOBS cohort previously [15]. To exclude the potential interference effect of T2D, we selected a study population consisting of 116 individuals with obesity but without T2D (mean ± SD: 46.8 ± 9.3 years; BMI: 42.7 ± 5.0 kg/m^2^) (Table 1) from the ongoing KOBS study [16]. Written informed consent was obtained from all participants, and the study protocol was approved by the Ethics Committee of the Northern Savo Hospital District (54/2005, 104/2008, and 27/2010) following the Helsinki Declaration.

**Table 1:** Characteristics of the donors of liver tissue from the cohort data used in the study

	Clinical characteristics
Total, N (male/female)	116 (27/89)
Age (years)	46.8 ± 9.3
BMI (kg/m^2^)	42.7 ± 5.0
fS-Total cholesterol (mmol/l), N = 110	4.3 ± 0.8
fS-HDL cholesterol (mmol/l), N = 110	1.1 ± 0.3
fS-LDL cholesterol (mmol/l), N = 109	2.5 ± 0.7
fS-Triglycerides (mmol/l), N = 109	1.3 (0.9–2.0)
fP-glucose (mmol/l)	5.8 ± 0.7
fS-insulin (mU/l), N = 115	14.7 (10.1–20.1)
Steatosis grade, N	
< 5%	62
5–33%	41
33–66%	6
> 66%	7
Lobular inflammation, N	19
Ballooning, N	18
Fibrosis stage, N	38

One-way ANOVA continuous variable or χ2 test. Data are present as mean ± SD. N- number of individuals, BMI- Body mass index, fS- fasting serum, fP- fasting plasma, HDL- high-density lipoprotein, LDL- low-density lipoprotein, SS-simple steatosis, NASH- non-alcoholic steatohepatitis

Liver biopsies were obtained during bariatric surgery and were scored for histology by an experienced pathologist according to the standard criteria, as previously described [17, 18]. Scoring criteria are summarized in Supplementary Table S1 and also described previously [19].

### Measurement of serum indole-3-propionic acid

Fasting serum samples were submitted to non-targeted LC–MS for metabolomics profiling (*n* = 116). The samples were analyzed with a UHPLC-qTOF-MS system (1290 LC, 6540 qTOF-MS, Agilent Technologies, Waldbronn, Karlsruhe, Germany), as previously described19. IPA was identified based on retention time and MS/MS spectral comparison with pure standard compound. IPA signal intensities (peak area) were considered for all further analyses [20].

### Liver RNA-sequencing and DNA methylation

Total RNA sequencing for the liver was performed using Illumina HiSeq 2500 and data was preprocessed following the previously described methodology [19, 21, 22]. We performed targeted differential expression analysis on transcripts affecting mitochondrial function/genesis using 1957 genes selected from the MitoMiner 4.0 database [23]. The liver DNA methylation analysis using Infinium HumanMethylation450 BeadChip (Illumina, San Diego, CA, USA) was performed as reported earlier along with the methodology [24, 25].

### Cell culture, TGF-β1 induction, and IPA treatment

Human hepatic stellate cells (LX-2) were kindly provided by Professor Stefano Romeo and were cultured and maintained (DMEM/F12; Biowest, L0093‐500, 1% Pen/Strep; Lonza, DE17‐602E, 2% FBS; Gibco, 10270‐106). To select the working dose of IPA, LX-2 cells were treated with several concentrations of IPA (10 µM, 100 µM, and 1 mM; Sigma, 220027) in DMEM/F12 for 24 h. Additionally, to investigate the capacity of IPA to inactivate HSCs, LX-2 cells were co-treated with 5 ng/ml TGF-β1 (R&D systems, 240‐B‐002/CF) and 1 mM IPA in serum-free media for 24 h. For the corresponding vehicle control, 4 nM HCL with 0.1% BSA was used for TGF-β1, 0.05% of DMSO for IPA treatment, and both together for the co-treatment.

### Flow cytometry analysis

#### FITC Annexin V and 7-AAD apoptosis assay

Apoptosis was evaluated with FITC Annexin V Apoptosis Detection Kit with 7-AAD (Biolegend, San Diego, CA, USA, cat: 640922) following the manufacturer’s instruction. Briefly, LX-2 (1 × 10^5^ cells/well) was cultured in 12-well plates overnight followed by treatment with either several doses of IPA, or IPA and TGF-β1. The next day, floating and attached cells were collected, trypsinized, washed with PBS, resuspended with Annexin V binding buffer, and incubated for 15 min with FITC Annexin V and 7-AAD.

#### Mitotracker™ red CMXRos mitochondrial activity assay

Mitotracker™ Red CMXRos (MTR) (Thermo Fisher Scientific, Carlsbad, CA) was used for staining mitochondria of live cells depending on oxidative activity. For the MTR assay, same density of LX-2 cells was incubated with IPA and TGF-β1. After 24 h, live cells were trypsinized, washed with PBS, and incubated with 100 µM of MTR in serum-free media at 37 °C for 20 min as previously described [26]. For live cell morphological analysis, cell size and cell cytoplasmic complexity were analyzed using forward scatter (FSC) and side scatter (SSC) parameters, respectively.

All data were acquired (30,000 events) with NovoCyte Quanteon (Agilent) and analyzed using NovoExpress^®^ Software 1.4.1 or FlowJo V.10 software.

### Mitochondrial respiration

Real-time measurements of oxygen consumption rate (OCR) and extracellular acidification rates (ECAR) were performed using the Seahorse Extracellular Flux Analyzer with Seahorse XF Cell Mito Stress (Agilent Technologies, Santa Clara, CA), following the manufacturer’s instructions. Briefly, 2 × 10^4^ LX-2 cells/well were seeded onto XF96 cell culture plates. After overnight incubation, cells were treated with IPA and TGF-β1 (Supplementary Methods 1). Data analysis was performed using Seahorse XF Wave software, including the Seahorse XF Cell Energy Phenotype Test Report Generator. The bioenergetic health index (BHI) was calculated accordingly [27].

### Quantitative RT-PCR & mtDNA copy number analysis

Total RNA was transcribed into cDNA as described elsewhere [15]. Human 60S acidic ribosomal protein P0 (*RPLP0*) and Cyclophilin A1 (*PPIA*) mRNA levels served as constitutive gene controls. The QuantStudio 6 pro-Real-Time PCR System (Thermo Fisher, Landsmeer, The Netherland) using TaqMan^™^ Fast Advanced Master Mix (Applied Biosystems) or Sensifast SYBR Lo‐ROX Kit (Bioline, BIO 94050) with comparative CT (ΔΔCT) cycling parameters and ^∆∆^Ct method was used to calculate relative fold gene expression. The primer details are demonstrated in Supplementary Tables S2 and S3.

The nuclear DNA (ncDNA) and mitochondrial DNA (mtDNA) were extracted using the DNeasy Blood & Tissue kit (Qiagen) as previously described [28]. The relative mtDNA amount was calculated by taking the ratio between each target mtDNA region and the geometric mean of three nuclear DNA regions (mtDNA / ncDNA), as summarized in Supplementary Methods 2. The primer details for mtDNA and ncDNA are shown in Supplementary Table S4.

### Laser-scanning confocal microscopy analysis

The inter- and intra-cellular mitochondrial networks were viewed by staining live cells with Mitotracker^™^ Red CMXRos (MTR) (Thermo Fisher Scientific, Carlsbad, CA). LX-2 cells (1 × 10^4^ cells/well) were cultured on appropriate glass-bottom culture plates on chamber slides (Ibidi GmbH, Martinsried, Germany). After 24 h, live LX-2 cells were incubated with 100 µM of MTR for 20 min at 37 °C, and cell nuclei were stained with DAPI (1 μg/ml, Sigma-Aldrich), as previously described [29]. The mitochondria network was visualized with 63 × NA 1.3 objective on a Zeiss Axio Observer inverted microscope equipped with a Zeiss LSM 800 confocal module (Carl Zeiss Microimaging GmbH, Jena, Germany) at 37 °C with humidified 5% CO_2_. We acquired ten Z-stack images from each sample type. Each Z-stack included 30 slices, with each slide having a thickness of 9.86 µm. For each sample, images of ten different fields were acquired with ZEN 2009 software (Carl Zeiss Microimaging GmbH, Jena, Germany), and mitochondrial morphology analysis was performed using ImageJ (v1.54d) software [30, 31] following the parameters detailed in Supplementary Methods 3.

### Ultrastructural analysis through transmission electron microscopy & phase contrast microscopy

Cells were fixed in 2% glutaraldehyde in 0.1 M phosphate buffer solution, followed by fixation in 1% osmium tetroxide solution (Sigma Aldrich; Missouri, USA), gradual dehydration in acetone (Merck, Darmstadt, Germany), and soaking in epon resin. Ultrafine cuts were obtained and counterstained with 1% uranyl acetate (Merck, Darmstadt, Germany) and 1% lead citrate (Merck, Darmstadt, Germany). Ultrastructural imaging was obtained by Transmission Electron Microscopy JEM 2100F EXII, (Jeol Ltd, Tokyo, Japan) at 80-kV acceleration voltage.

Cell morphology of LX-2 cells with IPA treatment after 24 h was analyzed by phase contrast microscopy using a Zeiss inverted light microscope (Zeiss Axio Vert.A1 and AxioCam MRm, Jena, Germany) at 50 × magnification.

### Statistical analysis

Clinical data are demonstrated as mean ± SD or median (interquartile range:IQR). One-way ANOVA continuous variable or χ^2^ test for the categorical variable was used to study the differences between the three study groups. False discovery rate (FDR) was used for multiple test correction, and the genes with FDR < 0.05 were considered statistically significant. Spearman correlation analysis was used to correlate DNA methylation of CpGs and IPA signal intensities and nominal p-values (p < 0.05) are presented.

Pathway analysis was done separately for 268 transcripts (nominal p < 0.01), 119 mitochondrial-related transcripts (nominal p < 0.05), and 4350 CpGs mapped to 3093 transcripts in the liver all of which were associated with circulating serum IPA levels using WEB-based Gene SeT Analysis Toolkit (WebGestalt). The freely available Venny DB (version 2.1.0) tool was used to find the overlapping genes and StringDB (version 11.5) was used to visualize the protein–protein interactions.

For LX-2 experiments, samples were tested for normality using D´Agostino-Pearson tests. Data were obtained from at least three biological replicates and One-way ANOVA followed by Bonferroni’s post hoc test was performed. Statistical significance was accepted at p < 0.05. Data are expressed as mean ± SD, and the number of experiments performed is indicated in each figure. All analyses and graphs were performed using the statistical software GraphPad Prism 8 for Windows (GraphPad Software Inc., version 8.4.3, San Diego, USA).

## Results

### Serum IPA levels are associated with apoptosis-related transcripts and DNA methylation in human liver

First, we investigated the association of serum IPA levels with liver global and mitochondrial-related transcripts. For the global transcript profile, the top gene associated with serum IPA levels was *MAPKAPK3* (FDR = 0.0077; mitogen-activated protein kinase activated protein kinase 3) and for the mitochondrial-related transcript profile was *AKT1* (FDR = 0.7621; AKT serine/threonine kinase 1) (Additional File 1 and Additional File 2).

**Fig. 1:**
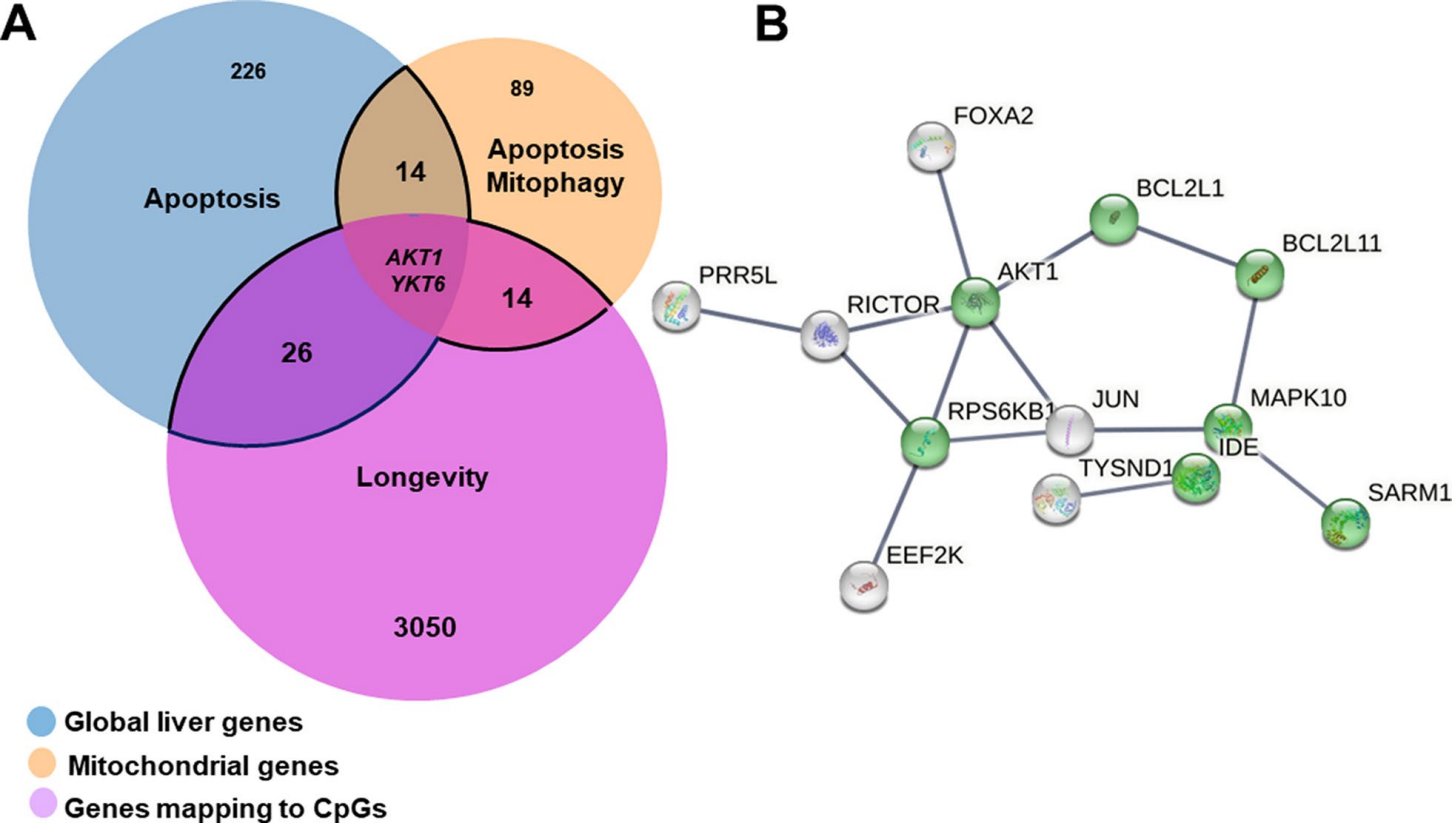
Overlap analysis of global transcripts, mitochondrial-related transcripts, and DNA methylation in the human liver associated with serum IPA levels. **A** The Venn diagram representing 268 global transcripts, 119 mitochondrial-related transcripts, and DNA methylation of transcripts that map to 3092 CpGs sites correlated with serum IPA levels (p-value < 0.01 for global and DNA methylation, p-value < 0.05 for mitochondrial-related transcripts). The main overlapped transcripts are shown in the middle (*AKT1* and *YKT6*). **B** Interaction map of 13 genes with the highest score interactions with other genes (0.900) using the StringDB online tool from the 56 overlapped genes (black lines area) that significantly associated with serum IPA levels. In green: genes that map to gene ontology (GO) cellular component: mitochondrion (GO:0005739). AKT1 is the protein that has more highest score interactions (0.900) with other proteins based on data support (based on text mining, experiments, databases, and co-expression). The network nodes represent proteins and the edges represent protein–protein associations

Next, we subjected the global (n = 268; p < 0.01) and mitochondrial-related transcripts (n = 119; p < 0.05) leading to the identification of apoptosis as the top canonical pathway (p = 0.0089). For mitochondrial-related transcripts associated with serum IPA levels, highlighted apoptosis (FDR = 0.00001), mitophagy (FDR = 0.00029), and TNF signaling pathway (FDR = 0.000006) (Fig. 1A, Table 2, and Supplementary Fig. 1A-B).

**Table 2:** Pathway enrichment analysis of global transcripts (p-value < 0.01), mitochondrial-related transcript (p-value < 0.05), and DNA methylation (p-value < 0.01) that correlated with circulating serum IPA signal intensity

List	Number of genes	Pathway	Enriched genes	q-value	p-value
Global	268	Apoptosis	*BCL2L11, BIRC2,MAPK10*	0.585	0.0089*
Mitochondrial	119	Apoptosis	*BCL2L1*	0.0017*	0.000010*
			*BCL2L11, BOK, MAPK10*		
		Mitophagy	*TNFRSF1A*	0.0095*	0.00029*
			*BCL2L1, JUN, MAPK10,*		
		TNF signaling pathway	*MFN1, TBC1D15,*	0.0017*	0.000006*
			*AKT1, DNM1L, JUN, MAP2K1, MAPK10, MAPK14, RIPK3, TNFRSF1A*		
CpGs	4350	Longevity	*RPS6KB1, AKT1S1, HSPA1L, SOD1, AKT1, PIK3CD, ADCY4, ADCY9*	0.224	0.006*
Overlapped Global + Mitochondrial + CpGs	56	Apoptosis	*BCL2L1, BCL2L11, MAPK10*	0.0185*	0.00029*

^*^p-value and q-value less than 0.05

As gut microbiota-derived metabolites can regulate epigenetic makeup via DNA methylation [32], we investigated whether serum IPA levels are associated with liver DNA methylation. We found that the top two methylation sites associated with serum IPA levels were close to proline and serine-rich 3 (*C19orf55*) and heat shock protein family B (small) member 6 (*HSPB6*) (Additional File 3). DNA methylation of 4350 CpGs (p < 0.01) associated with serum IPA levels were enriched for longevity regulating pathway (p = 0.006) (Fig. 1A, Table 2, and Supplementary Fig. 1C).

To understand the biological mechanisms related to the association between serum IPA levels, global, mitochondrial-related transcripts, and DNA methylation in the human liver, we overlapped the genes identified in the previous pathway analysis (Fig. 1A). The enriched pathway analysis from 56 overlapped genes (within the black lines in Fig. 1A) emphasized the apoptosis pathway (p = 0.00029) revealing two genes to be common in all three analyses: *AKT1* and *YKT6* (YKT6 v-SNARE homolog) shown in the Venn diagram (Supplementary Fig. 2 and Fig. 1A). Interestingly, we found a positive association of *AKT1* (cg19831386) and *YKT6* (cg24161647) with serum IPA levels (Additional File 3). To identify potential protein–protein interactions between the gene products, 13 of 56 overlapped genes with the highest score (0.900) of the shared area were used as input to build an interactive map. Based on the confidence level (edge confidence), the topmost interactive gene was *AKT1* with the highest score (0.900) (Fig. 1B).

### IPA induces apoptosis in LX-2 cells

**Fig. 2:**
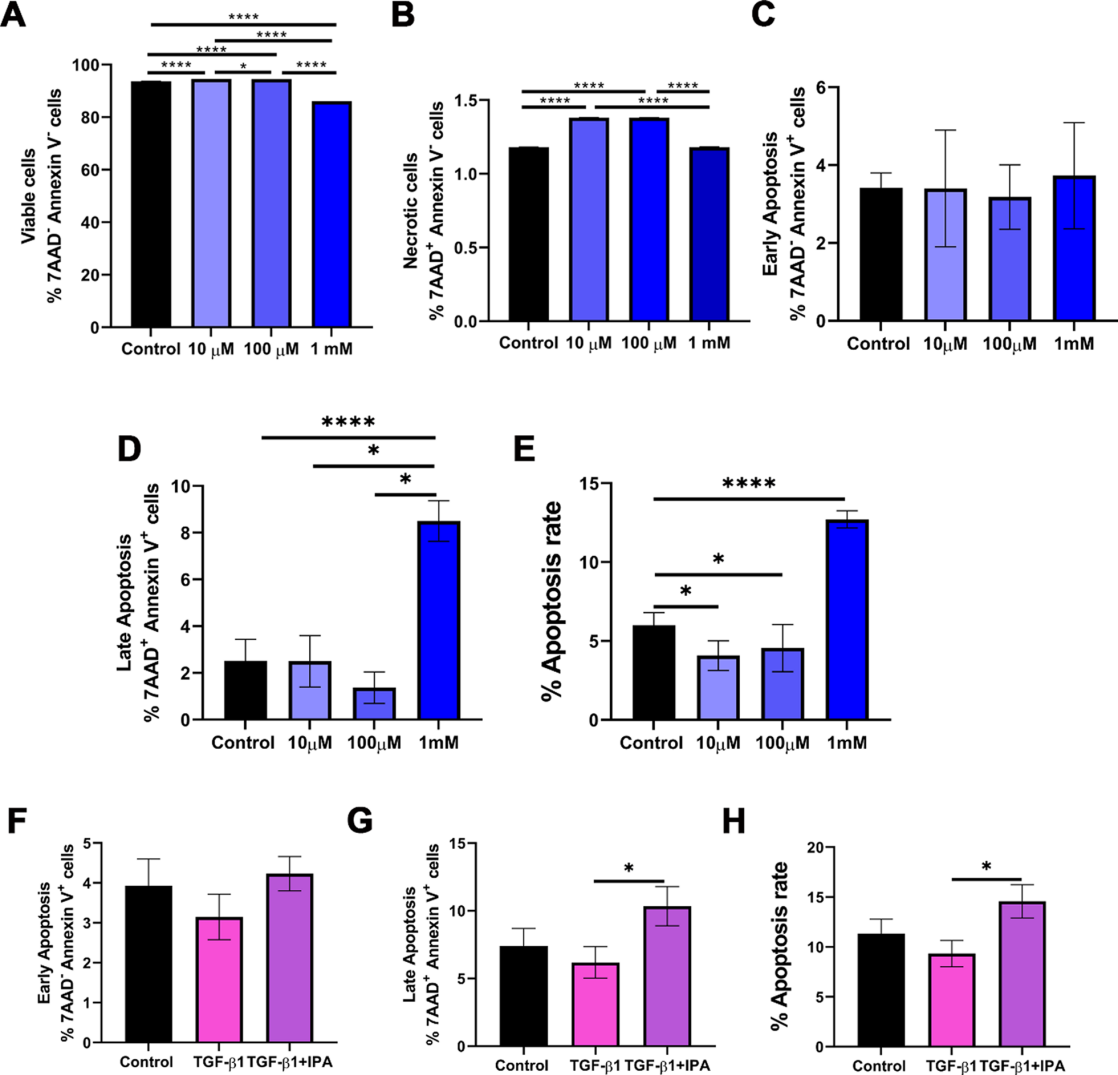
IPA induces apoptosis in LX-2 cells. Quantification of apoptosis rate and cell morphology was performed on flow cytometry analysis using a double staining method with Annexin V and 7-AAD.** A**-**D** Cells were incubated with 10 µM, 100 µM, and 1 mM of IPA for 24 h or **F**–**H** TGF-β1 (5 ng/ml) and 1 mM of IPA in non-serum media for 24 h. **A** Viable cells (Annexin V ^−^/ 7AAD^−^), **B** Necrotic cells (Annexin V ^−^/ 7AAD^+^),** C**, **F** Early (Annexin V ^+^/ 7AAD^−^), **D**, **G** Late (Annexin V ^+^/ 7AAD.^+^), and **E**, **H** the total of early and late apoptotic cells as the percentage of apoptosis rate (%). Data were shown as mean ± SD, n = 3 independent experiments. One-way ANOVA with Bonferroni´s post hoc test was used for statistical comparisons. *p < 0.05; ****p < 0.0001

Based on our pathway analyses demonstrating apoptosis as the main pathway, we investigated whether IPA treatment affects HSCs apoptosis in vitro. We have previously shown that doses of IPA (10 µM, 100 µM, and 1 mM) were non-toxic in LX-2 cells [15]. Here we showed that the treatments of 10 and 100 µM have an increase of viable and necrotic cell rates. However, at 1 mM IPA concentration, cell viability decreases without change in necrotic cell rate compared to control (Fig. 2A, B). Next, to discover the best concentration to induce apoptosis in LX-2 cells, we tested 10 µM, 100 µM, and 1 mM IPA for 24 h (Fig. 2A–E and Supplementary Fig. 3A-B). Interestingly, IPA 10 µM and 100 µM decreased apoptosis rate (%), however, IPA 1 mM increased the late apoptosis and apoptosis rate (%) compared to control and was chosen for further experiments (Fig. 2A–D).

As we previously showed, TGF-β1 5 ng/ml was able to induce HSC activation by increasing the gene expression of classical markers [15]. LX-2 cells were co-treated with TGF-β1 5 ng/ml and IPA 1 mM (Fig. 2E–H). TGF-β1 treatment did not change the apoptosis rate, however, the co-treatment with IPA increased the late apoptosis and apoptosis rate (%) compared to TGF-β1 treatment (Fig. 2E–H). These findings demonstrated that IPA 1 mM could promote apoptosis on LX-2 cells regardless of TGF-β1 induction.

### IPA decreases mitochondrial respiration and induces a less energetic profile in LX-2 cells

**Fig. 3:**
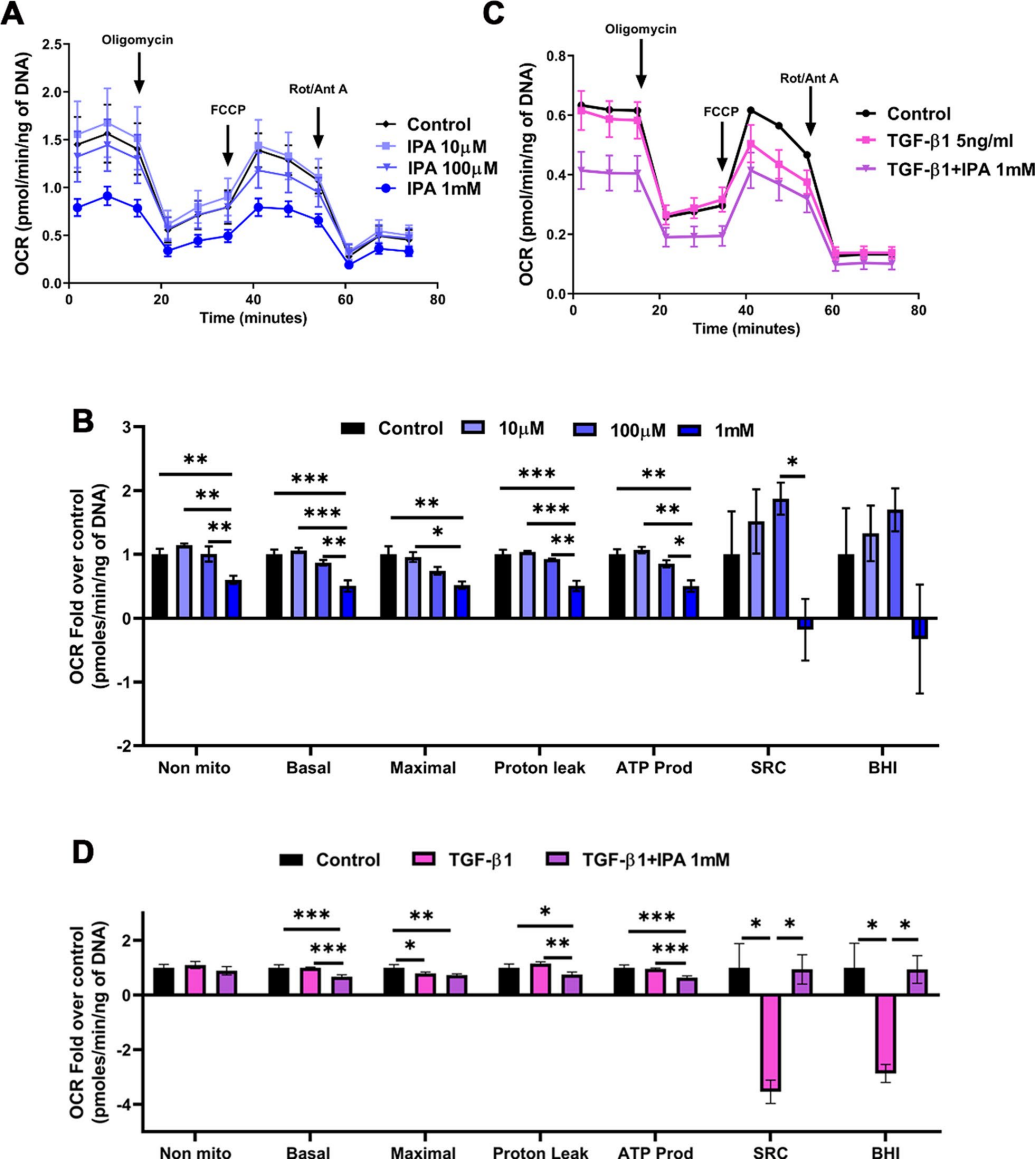
IPA decreased mitochondrial respiration in LX-2 cells. Mitochondrial respiration curves (OCR) are presented as parameters of mitochondrial respiration (non-mitochondrial respiration, basal respiration, maximal respiration, proton leak, ATP production, SRC, and BHI). **A**, **B** Cells were incubated with 10 µM, 100 µM, and 1 mM of IPA for 24 h. **C**, **D** Cells were incubated with TGF-β1 (5 ng/ml) and 1 mM of IPA in non-serum media for 24 h. All the measurements were normalized for the DNA amount by CyQuant kit. BHI: Bioenergetic health index, SRC: Spare respiratory capacity OCR: Oxygen consumption rate. Data were shown as mean ± SD, n = 5 independent experiments. One-way ANOVA with Bonferroni´s post hoc test was used for statistical comparisons. *p < 0.05; **p < 0.01; and ***p < 0.001

Next, we investigate the effects of IPA on mitochondrial respiration in LX-2 cells. Our results demonstrated that 1 mM IPA decreased the oxygen consumption rate (OCR) parameters: non-mitochondrial, basal and maximal respiration, proton leak, and ATP production (Fig. 3A, B), without changing the bioenergetic health index (BHI), compared to control.

To gain deeper insights into the impact of IPA on the bioenergetics profile in TGF-β1-activated LX-2 cells, we analyzed the mitochondrial oxidative phosphorylation by OCR (Fig. 3C, D). The results showed that TGF-β1 treatment was able to decrease maximal respiration, spare respiratory capacity (SRC), and BHI when compared to the control (Fig. 3C, D). Moreover, the co-treatment decreased basal respiration, proton leak, and ATP production, but SRC and BHI were significantly higher when compared to the TGF-β1 treatment (Fig. 3C, D).

We additionally conducted a “Cell Energy Phenotype Test” provided by the Seahorse software (Supplementary Fig. 4A-D). As shown in Supplementary Fig. 3B, metabolic potentials for OCR and ECAR were both reduced after TGF-β1 treatment, however, no differences were observed in co-treatment and only IPA treatment when compared to control. Furthermore, a decrease in baseline and stress levels for OCR after co-treatment and only IPA treatment compared to control was recorded (Supplementary Fig. 4C). Interestingly, a similar pattern was observed for co-treatment when compared with TGF-β1 treatment without change at baseline and stressed levels for ECAR (Supplementary Fig. 4C). The decrease in mitochondrial oxidative phosphorylation and the ability of the co-treatment to restore SCR and BHI from the effects of TGF-β1 treatment was seen without changing the metabolic potential (OCR and ECAR) in HSCs. Altogether, these results demonstrated that IPA could decrease the bioenergetics in HSCs, suggesting that IPA can induce a less energetic profile, shifting HSC phenotype towards inactivation (Supplementary Fig. 4D).

### IPA alters mitochondrial dynamics and mtDNA amount in LX-2 cells

**Fig. 4:**
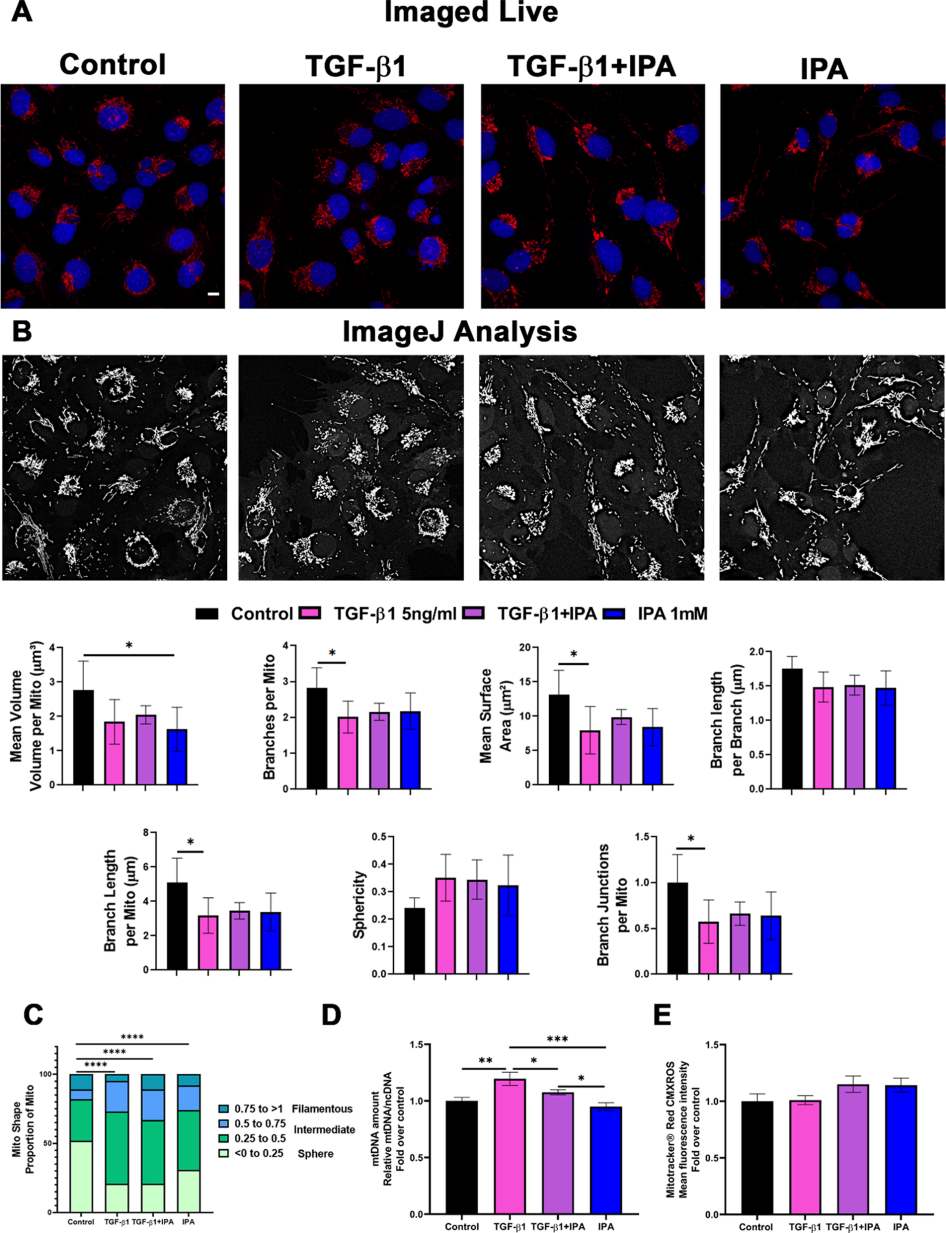
IPA alters mitochondrial dynamics and mtDNA amount in LX-2 cells. **A** Representative confocal images of live LX-2 cells with TGF-β1 (5 ng/ml) and IPA 1 mM in non-serum media for 24 h showing the mitochondrial network stained by Mitotracker™ Red CMXRos and blue nucleus with DAPI. All data were presented with at least 15 images per group. We acquired 10 Z-stack images from each sample type. Each Z-stack included 30 slices, with each slide having a thickness of 9.86 µm. Scale bar, 10 µm. **B** Representative objects identified by application of adaptative thresholding to the images (only mitochondria). Quantitative analysis and comparison of morphological mitochondrial network connectivity were performed on all cells in each group. **C** Frequency of proportion of mito shape. Values close to 0 mean spheres and close to 1 mean filamentous. **D** The mitochondria DNA amount (mtDNA) was measured as previously described in Material and Methods. **E** Mitotracker^™^ Red CMXRos analyses were performed by flow cytometry (30.000 events) as previously described in Material and Methods. Data were shown as mean ± SD, n = 3 independent experiments. One-way ANOVA with Bonferroni´s post hoc test was used for statistical comparisons. *p < 0.05; **p < 0.01 ***p < 0.001; and ****p < 0.0001

The effect of IPA on mitochondrial dynamics was viewed in the 3D quantification of mitochondrial morphology and network connectivity with MTR staining (Fig. 4 and Supplementary Fig. 5). In Fig. 4, TGF-β1 treatment decreases mean surface area, number of branches, total branch length, and branch junctions (Fig. 4A and B) and changes the proportion of mitochondria from spherical towards intermediate morphology (Fig. 4C) versus control. Only IPA treatment decreased mitochondrial mean volume and changed the proportion of mitochondria from spherical towards intermediate morphology compared to control (Fig. 4A). In contrast, the sphericity, mean branch length, and the mitochondrial activity assessed with mitochondrial membrane potential-dependent MTR (Fig. 4A and E) remained constant and no changes were seen in these parameters between the groups. In summary, these results demonstrated that TGF-β1 and IPA treatment seemed to modulate the mitochondrial shape and size together with the complexity of the network in LX-2 live cells.

Next, we analyzed mtDNA amount as a read-out for mitochondria number in LX-2 cells. TGF-β1 treatment increased the mtDNA amount when compared with the control (Fig. 4D). While, co-treatment demonstrated a decrease in mtDNA amount when compared with TGF-β1 treatment (Fig. 4D), showing that IPA may decrease the mtDNA and possible mitochondria number together with the mitochondrial respiration (Fig. 3C). Additionally, IPA seems to decrease mtDNA amount in co-treatment treatment without affecting the mitochondrial activity by MTR (Fig. 4A–C).

### IPA can modulate the expression of genes regulating fibrosis, apoptosis, survival, and mitochondrial dynamics in LX-2 cells

**Fig. 5:**
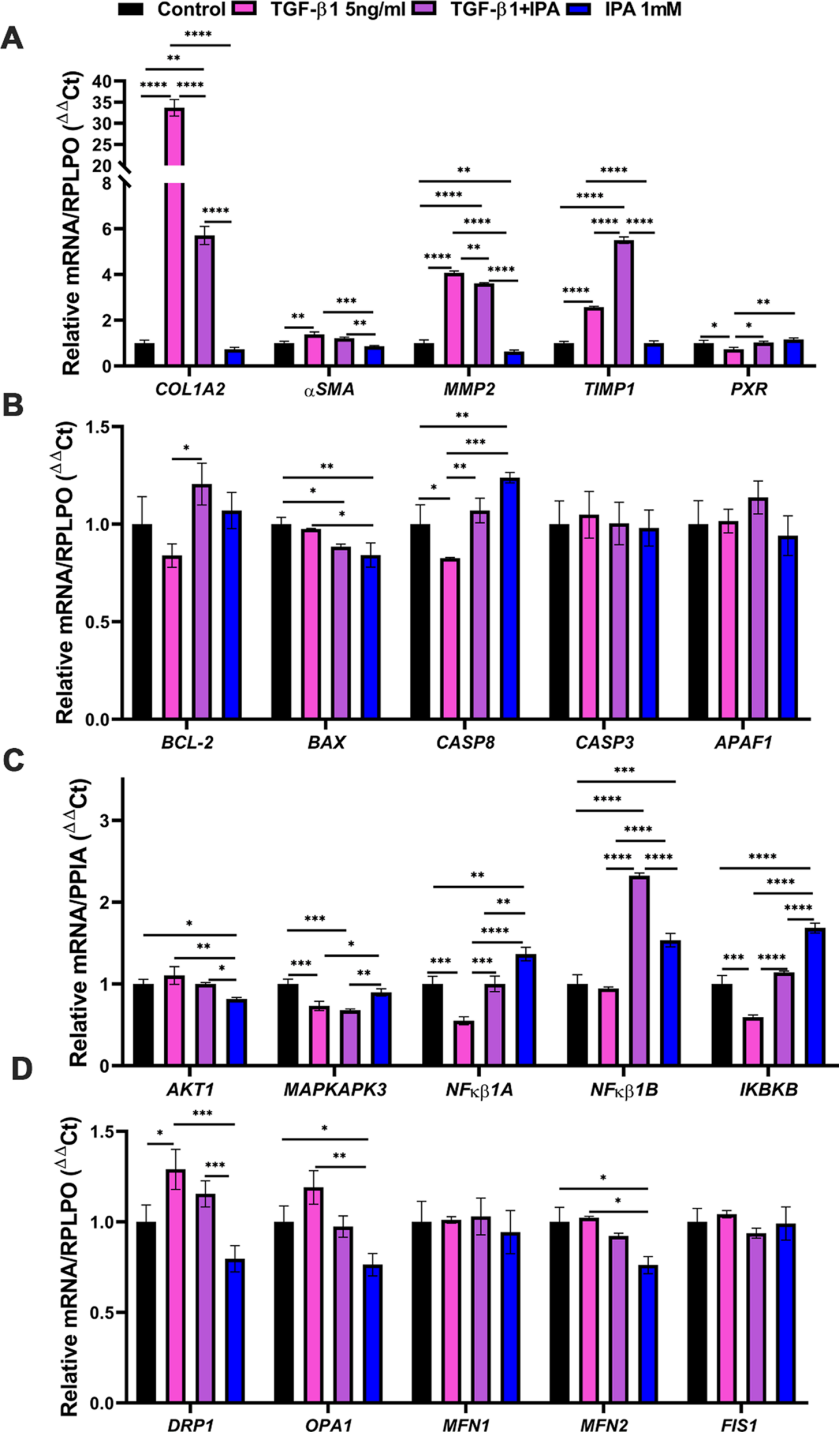
IPA can modulate the fibrogenic, apoptotic, survival, and mitochondrial dynamics gene expression in LX-2 cells. Bar plots represent the relative mRNA to endogenous controls (*RPLP0* or *PPIA*) after the induction of LX-2 cells with TGF-β1 and IPA in non-serum media for 24 h. **A** Fibrogenic, **B** apoptotic, **C** survival genes, and **D** mitochondrial dynamics gene expression. Data were shown as mean ± SD, n = 3 independent experiments. One-way ANOVA with Bonferroni´s post hoc test was used for statistical comparisons. *p < 0.05; **p < 0.01; ***p < 0.001; and ****p < 0.0001

We examined the association of IPA with mRNA levels of genes related to fibrogenesis, apoptosis, survival, and mitochondrial dynamics in LX-2 cells (Fig. 5A–D). TGF-β1 treatment showed increased expression of genes such as collagen type I alpha 2 chain (*COL1A2*)*,* α-smooth muscle actin (*αSMA*)*,* matrix metalloproteinase-2 (*MMP2*), tissue inhibitor of metalloproteinase 1 (*TIMP1*), and dynamin 1-like (*DRP1*), when compared to control, demonstrating increased fibrogenesis and activation. Additionally, TGF-β1 treatment was able to decrease the mRNA levels of nuclear receptor pregnane X receptor (*PXR*), Caspase-8 (*CASP8*), *MAPKAPK3*, nuclear factor of kappa light polypeptide gene enhancer in B-cells inhibitor, alpha (*NFκB1A*), and inhibitor of nuclear factor kappa B kinase subunit beta (*IKBKB*) when compared to control (Fig. 5A–D). The co-treatment with TGF-β1 and IPA decreased the expression of *COL1A2 and MMP2*, however, increased the mRNA levels of *PXR*, *TIMP1*, B-cell lymphoma-2 (*BCL-2*), *CASP8*, *NFκB1A, NFκB1-beta*, *IKBKB* when compared with TGF-β1 treatment. IPA treatment significantly decreased *MMP2,* Bcl-2 Associated X-protein (*BAX*), *AKT1*, optic atrophy protein 1 (*OPA1*), and mitofusin 2 (*MFN2*) expression, nonetheless, *CASP8*, *NFκB1A, NFκB1B, IKBKB* expression increased compared to control. However, no differences were found in Caspase-3 (*CASP3*), apoptotic peptidase activating factor 1 (*APAF1*), mitofusin 1 (*MFN1*), and fission 1 (*FIS1*)*.* Altogether, these results show that IPA treatment modulates the expression of genes related to fibrogenesis, apoptosis, survival, and mitochondrial dynamics. Our data suggests that IPA treatment can reduce fibrogenesis in LX-2 cells; meanwhile promoting the survival stimulus to shift the phenotype towards inactivation.

### IPA changes cell morphology in LX-2 cells

**Fig. 6:**
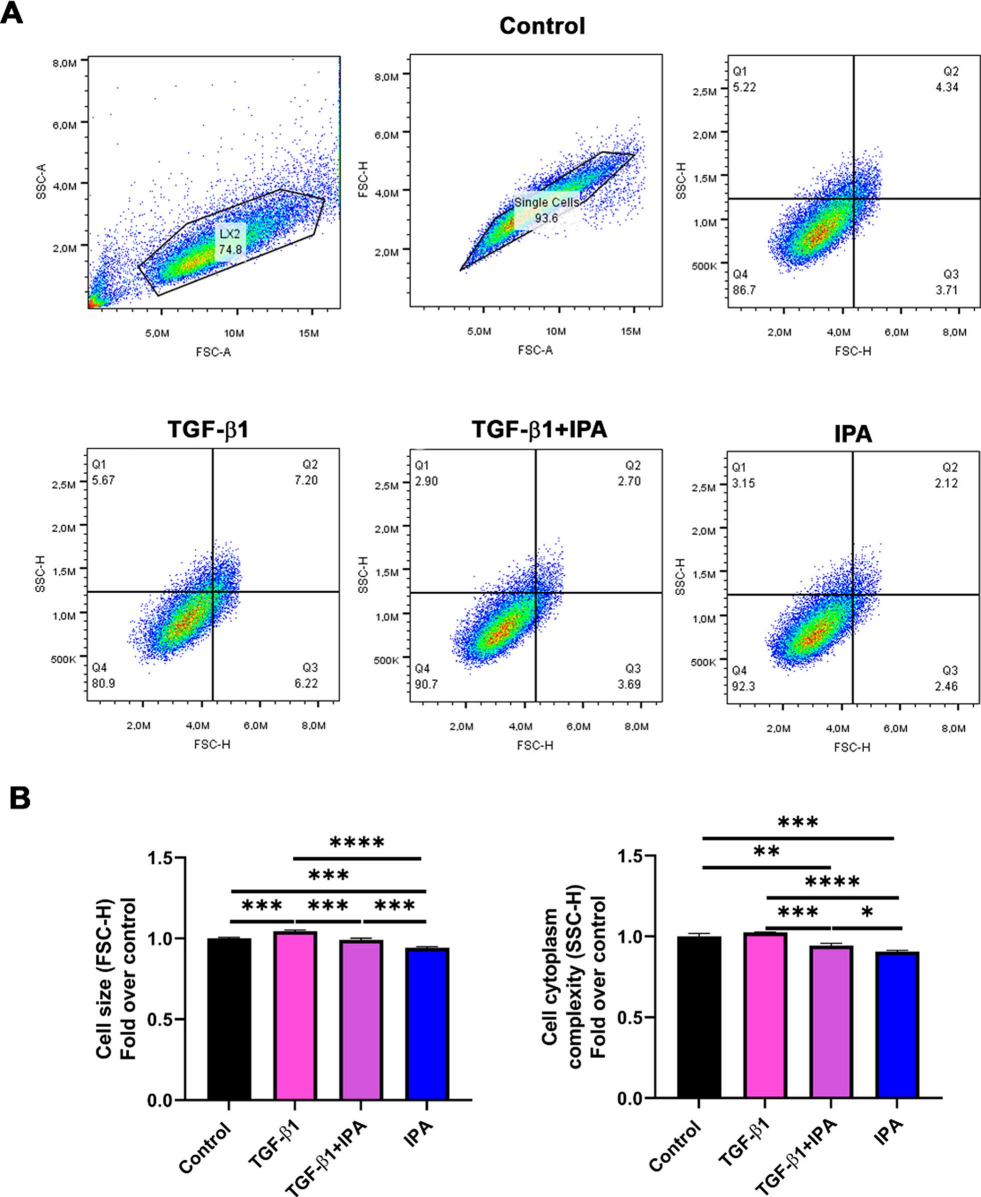
IPA changes cell size and cytoplasm complexity in LX-2 cells.** A** Representative images from flow cytometry analysis. The analysis was performed using a gating strategy for LX-2 cells: SSC-A/FSC-A determination of cell population, FSC-H/FSC-A doublet discrimination, and SSC-H/FSC-H for cell size and cell complexity analysis. Cells were incubated with TGF-β1 (5 ng/ml) and 1 mM of IPA in non-serum media for 24 h. LX-2 cells were gated into lower left quadrants (SSC-H^−^/FSC-H^−^), upper left (SSC-H^+^/FSC-H^−^), lower right (SSC-H^−^/FSC-H^+^), and upper right quadrants (SSC-H^+^/ FSC-H.^+^) for analysis of cell size and cell cytoplasm complexity. **B** Analysis of cell morphology for FSC-H (forward scatter, cell size) and SSC-H (side scatter, cytoplasmic complexity) by flow cytometry (30.000 events). Data were shown as mean ± SD, n = 3 independent experiments. One-way ANOVA with Bonferroni´s post hoc test was used for statistical comparisons. *p < 0.05; **p < 0.01; ***p < 0.001 and ****p < 0.0001

Thereafter, changes in cell size (FSC-H), cell cytoplasm complexity (SSC-H) by flow cytometry (Fig. 6A, B), and changes in cell morphology by transmission electron microscopy (TEM) and phase contrast microscopy after IPA treatment were assessed (Supplementary Fig. 6A-B). As expected, TGF-β1 treatment increased the cell size when compared with the control (Fig. 6A, B), showing classical rough endoplasmic reticulum dilated (ER*) and phagolysosomes (P), suggesting HSC activation (Supplementary Fig. 6A). However, co-treatment with TGF-β1 and IPA decreased cell size, cell cytoplasm complexity (Fig. 6A, B), and ER* content when compared to TGF-β1 treatment (Supplementary Fig. 6A). Additionally, IPA treatment decreased cell size, cell cytoplasm complexity (Fig. 6A, B), P, and ER* content compared to the control (Supplementary Fig. 6A). Furthermore, the apoptotic cell content increased after IPA treatment for 24 h when compared with the control (white arrows Supplementary Fig. 6B). Altogether, these results suggest that IPA 1 mM could stimulate apoptosis in HSCs and reverse the TGF-β1-induced changes in the cell morphology parameters, modulating the cell size and complexity that could be related to the HSCs inactivation.

## Discussion

Gut-derived metabolites like IPA become a research hotspot showing that new targets can be found in the gut microbiome. Thus, it was interesting that IPA, a metabolite that we have linked with liver fibrosis in humans [15] has been demonstrated as a potential anti-fibrotic compound based on animal models [13, 14]. Here, we demonstrate for the first time the association of serum IPA with liver global transcriptomics and DNA methylation in obese individuals without T2D highlighting apoptosis, mitophagy, and longevity together with a possible candidate gene *AKT1* for regulating liver homeostasis. Another novelty of our study is that we demonstrated the interaction of IPA treatment in LX-2 cells with apoptosis, cell morphology, mitochondrial bioenergetics and dynamics, suggesting a less energetic profile, shifting HSC phenotype towards inactivation, rendering IPA a potential candidate for the resolution of liver fibrosis.

We found that apoptosis, mitophagy and longevity were the top canonical pathways enriched by liver genes associated with circulating serum IPA. A disturbed mitochondrial quality control (MQC) system leads to mitochondrial dysfunction, mitophagy, and apoptosis which contributes to the development of MASLD [33, 34]. In this way, we can suggest that IPA could be involved in the maintenance of cellular dynamics and mitochondrial integrity via apoptosis, mitophagy and longevity in the liver. Our data revealed two genes to be common in all three analyses: *YKT6* and *AKT1*. Interestingly, *YKT6* is a SNARE protein involved in the process of cell membrane fusion that plays a role in autophagy and mitophagy forming a priming complex with STX17 and SNAP29 on autophagosomes, which promotes the fusion of autophagosomes with lysosomes [35]. Additionally, loss of YKT6 function can lead to impaired mitophagy [36] and the upregulation of YKT6 is related to the progression of hepatocellular carcinoma (HCC), suggesting increased cell survival [37]. AKT1 on the other hand is the topmost interactive gene that plays an important role in the PI3K/AKT signaling pathway, cell cycling, cell migration, proliferation, focal adhesion, mitochondrial function, and collagen secretion in liver diseases [38–40]. When activated, the PI3K/AKT signaling pathway can activate the HSCs, cells responsible for ECM production, and the dysregulation can contribute to the onset and progression of liver fibrosis [40]. Furthermore, AKT is one of the key cell survival factors that can repress p53-dependent apoptosis or AKT activation can be associated with inhibition of apoptosis in the liver [41, 42]. These results suggest that IPA could be linked to mitochondrial-associated apoptosis in the liver, affecting liver cells' decisions between entering apoptosis or survival. These actions could be regulated by AKT and/or YKT6 as candidate genes, being crucial for liver homeostasis.

Our results showed that 1 mM of IPA induced apoptosis and decreased the mitochondrial respiration in LX-2 cells regardless of TGF-β1 treatment. Importantly, apoptosis is the major pathway to eliminate fibrogenic and activated HSCs, being a critical point toward the physiological response to the reversibility of liver fibrosis [4, 43]. Additionally, the restoration of BHI observed in LX-2 cells after the cotreatment provides new insights into the potential effect of IPA to modulate mitochondrial bioenergetics. HSCs in a quiescent and inactive state generally rely on mitochondrial oxidative phosphorylation for ATP production and are metabolically less active. On the other hand, the activation of HSCs increases mitochondrial respiration and biogenesis to compensate for the energy demands shifting into a glycolytic state [44]. Indeed, IPA did not affect the metabolic potential and ECAR suggesting that the glycolytic pathway was less preferred. Similarly, another study presents that IPA 1 mM was able to modulate the mitochondrial respiratory chain activity in cardiomyocytes, human hepatic cell line (Huh7), and human umbilical vein endothelial cells (HUVEC), however, no effects of IPA on glycolysis were found in cardiomyocytes, suggesting that IPA can affect the bioenergetics of other cell types [45]. In this sense, we suggest that IPA 1 mM can act as a mild chemical uncoupling agent based on the significant decrease in the expression of fibrogenic genes, cell morphology, and mitochondrial bioenergetics without changing the mtDNA amount [46]. Mitochondrial uncouplers can inhibit culture-induced fibrogenesis and HSC activation [47], and reduce mitochondrial ATP production regulated or induced by some proteins like uncoupling proteins (UCPs) or adenine nucleotide translocases (ANTs). This phenomenon can protect the cells against apoptosis and/or promote it, according to cell type [46]. However, further studies are needed to elucidate the function of IPA as a mitochondrial uncoupler in HSC inactivation.

Next, we explored whether changes in mitochondrial respiration were reflected in mitochondrial morphology in live LX-2 cells. Interestingly, TGF-β1 treatment changed the proportion of mitochondria from sphere to intermediate along with reduced mitochondrial branching and increased expression of *DRP1*, a key factor in mitochondrial fission [48]. Moreover, mitochondrial fragmentation is associated with the overall network complexity and the shift from fusion to fission is essential for HSC activation while inhibiting mitochondrial fission causes HSC apoptosis [49]. Thus, our results suggest that TGF-β1 treatment could induce a less complex mitochondrial network with fewer branching which is more typical for mitochondrial fission associated with activated HSCs. Further, our data showed that IPA could change the proportion of mitochondria from sphere to intermediate decreasing *OPA1* and *MFN2* expression. Reduced OPA1 activity has been shown to induce a mitochondrial membrane potential reduction and trigger apoptosis [50]. *MFN2* is known to mediate mitochondrial fusion and apoptosis [51]. These results demonstrated that LX-2 cells induced by TGF-β1 and/or IPA treatment seem to modulate the mitochondrial shape and size together with the activation state and complexity of the network.

Our results illustrate that cotreatment with TGFβ-1 and IPA decreases mtDNA and cell morphology parameters by modulating the mRNA expression of genes related to fibrogenesis, apoptosis, and survival in the cells that escaped from apoptosis. Indeed, IPA decreases the mRNA expression level of *AKT1* and important fibrogenic genes like *COL1A2* and *MMP2* but increases the apoptosis-related *CASP8*. Our results demonstrated reduced *BAX* and increased *TIMP1*, *BCL-2,* and NF-kB family subunit mRNA expression after IPA treatment, suggesting that IPA can stimulate the survival signals of HSCs escaping apoptosis. These molecules can be pro-survival signals in activated HSCs which may result from the enhanced expression of antiapoptotic proteins such as Bcl-2 and downregulation of pro-apoptotic BAX together with a complex interplay between TIMPs and NF-κB [5, 7]. IPA mediates its effects through PXR and we showed that *PXR* mRNA expression levels increase with cotreatment with TGF-β1 and IPA, proposing an inhibition of HSC activation. Activated PXR signaling is known to inhibit HSC activation in vivo and in vitro [52, 53]. Our results suggest that IPA could be involved in the clearance of activated HSCs by stimulating apoptosis, reducing fibrogenesis and mitochondrial metabolism but also enhancing the survival signals, processes typical for shifting the activated HSC phenotype towards inactivation. Another possible explanation for the potential mechanism and role of IPA in apoptosis is predominantly through the elimination of dysfunctional mitochondria by mitophagy (intrinsic pathway) and the extrinsic pathway by TNF signaling pathway (Table 1) that is directly linked with NF-κB survival signaling pathway (Supplementary Fig. 7). Interestingly, the enriched genes associated with IPA can induce both proapoptotic and pro-survival signaling in the apoptosis pathway [54] suggesting that IPA may induce the apoptosis pathway or survival through the interaction with those genes. However, how IPA may induce apoptosis or survival in HSC activation and the mechanistic pathways is still unknown.

IPA is a microbial metabolite derived from dietary tryptophan via the gut microbiome. It has been shown to exhibit anti-inflammatory, antioxidant, and epigenetic regulatory properties within the gut environment [55]. Studies suggest that IPA can modulate gut barrier function and reduce oxidative stress, which may contribute to its local physiological effects [56]. Indeed, IPA is transported to target organs through blood circulation and because IPA has a similar base metabolite structure to e.g. tryptophan, serotonin, and indole derivatives there is a metabolic effect of IPA, leading to competitive metabolic fates [52]. IPA may compete with tryptophan-derived metabolites for binding sites on enzymes or receptors, potentially disrupting normal metabolic pathways. This highlights the need for further research into its pharmacokinetics and pharmacodynamics to better understand its therapeutic window [57]. Whether this could also be happening in HSCs needs to be further elucidated.

We acknowledge that our study has some limitations. Exclusion of patients with T2D was performed to specifically search for IPA-related associations. We acknowledge that this limits the broader applicability of our results to patients with T2D and advanced liver disease. Although IPA physiological serum concentration in humans is 1-10 µM [11, 20], the 1 mM of IPA was chosen based on the highest non-toxic concentration [15] and the highest apoptosis rate with no differences in the percentage of necrotic cell population. Though supraphysiological IPA levels were used in this study, there is currently no consensus about the effective dose of IPA [52]. While the findings in our study are significant, the broader metabolic fate of IPA remains an area of active investigation. Additionally, our results of the association of serum IPA levels from liver transcripts together with DNA methylation are from liver tissue and not only HSCs. We chose to use human LX-2 cells based on our earlier findings that IPA is associated with HSC activation using transcriptomics analysis [15] and because HSCs are the main cells involved in the progression of liver fibrosis. The liver consists of many cell types, then in this sense other cell models like hepatocyte-HSCs-immune cells co-culture systems together with the caspase activation and DNA fragmentation, and the mechanism of action including protein levels should be considered to study the effects of IPA and the interaction between other hepatic cell types.

In conclusion, we demonstrated that IPA serum levels are associated with the expression of liver genes highlighting apoptosis, mitophagy, and longevity pathways. To our knowledge, our study provides for the first time new insights into the distinct IPA-induced effects on apoptosis and mitochondrial metabolism in HSCs being a novel compound for the resolution of activated HSCs. We believe that these results could bring a new understanding on the effects of human gut microbiome compounds that can develop new therapies for patients who suffer from chronic liver disorders. Future studies are required to understand how IPA can affect liver cells' and HSCs' decisions between entering apoptosis or survival and the molecular mechanism of action in the resolution of hepatic fibrosis.

## Supplementary Information

Additional file 1.

Additional file 2.

Additional file 3.

Additional file 4.

## Data Availability

All data is available within the manuscript and in the supplementary information.

## Abbreviations

AKT1: Serine/threonine kinase 1
APAF1: Apoptotic peptidase activating factor 1
BAX: Bcl-2 associated X-protein
BCL2: B-cell lymphoma-2
BMI: Body mass index
C19orf55: Proline and serine-rich 3
CASP3: Caspase-3
CASP8: Caspase-8
COL1A2: Collagen type I alpha 2 chain
CpGs: CpG sites
DRP1: Dynamin 1 like
ECAR: Extracellular acidification rates
FCCP: Carbonyl cyanide-p-trifluoromethoxyphenylhydrazone
FDR: False discovery rate
FIS1: Fission, mitochondrial 1
GAPDH: Glyceraldehyde-3-phosphate dehydrogenase
HSC: Hepatic stellate cell
HSPB6: Heat shock protein family B (small) member 6
IKBKB: Inhibitor of nuclear factor kappa B kinase subunit beta
KEGG: Kyoto encyclopedia of Genes and Genomes
KOBS: Kuopio OBesity Surgery
LX-2: Human hepatic stellate cells
MASLD: Dysfunction-associated Steatotic liver disease
MAPKPKA3: Mitogen-activated protein kinase-activated protein kinase 3
MFN1: Mitofusin 1
MMP2: Matrix metalloproteinase-2
MTR: Mitotracker Red CMXRos
NAFLD: Non-alcoholic fatty liver disease
NASH: Non-alcoholic steatohepatitis
NFκB1A: Nuclear factor of kappa light polypeptide gene enhancer in B-cells inhibitor, alpha
NFκB1B: Nuclear factor of kappa light polypeptide gene enhancer in B-cells inhibitor, beta
OCR: Oxygen consumption rate
OPA1: Optic atrophy protein 1
PVDF: Polyvinylidene fluoride
PXR: Nuclear receptor pregnane X receptor
RPLP0: Human 60S acidic ribosomal protein P0
SD: Standard deviation
SS: Simple steatosis
T2D: Type 2 diabetes
TGF-β1: Transforming growth factor beta 1
TIMP1: Tissue inhibitor of metalloproteinase 1
YKT6: V-SNARE homolog
αSMA: α-Smooth muscle actin
