# Supplementary material for: Indole-3-propionic acid promotes hepatic stellate cells inactivation
Source: J Transl Med. 2025 Mar 1;23:253. doi: 10.1186/s12967-025-06266-z (PMC11871697; doi:10.1186/s12967-025-06266-z)
Supplement: Supplementary file 1 — Additional file 1. [file 12967_2025_6266_MOESM1_ESM.docx]

**Indole-3-Propionic Acid Promotes Hepatic Stellate Cells Inactivation**

Mariana Ilha^1,2^, Ratika Sehgal^1,3^, Johanna Matilainen^4^, Kirsi Rilla^4^, Dorota Kaminska^1,5^, Shrey Gandhi^6,7^, Ville Männistö^8^, Charlotte Ling^9^, Stefano Romeo^10^, Päivi Pajukanta^11,12^, Eija Pirinen^13,14,15,16^ Kirsi A. Virtanen^17^, Kirsi H. Pietiläinen^18,19^, Maija Vaittinen^1*^, Jussi Pihlajamäki^1,20^

^1^Institute of Public Health and Clinical Nutrition, Department of Clinical Nutrition, University of Eastern Finland, Kuopio, Finland; ^2^Department of Neurosurgery, University of Pennsylvania, Philadelphia, USA; ^3^Department of Experimental Diabetology, German Institute of Human Nutrition Potsdam-Rehbruecke (DIfE), 14558 Nuthetal, Germany; ^4^Institute of Biomedicine, School of Medicine, Faculty of Health Sciences, University of Eastern Finland; ^5^Department of Medicine, Division of Cardiology, UCLA, Los Angeles, CA, USA; ^6^Institute of Immunology, University of Münster, Münster, Germany; ^7^Department of Genetic Epidemiology, Institute of Human Genetics, University of Münster, Münster, Germany; ^8^Departments of Medicine, University of Eastern Finland and Kuopio University Hospital, Finland; ^9^Epigenetics and Diabetes Unit, Department of Clinical Sciences, Lund University Diabetes Centre, Scania University Hospital, Malmö, Sweden; ^10^Department of Molecular and Clinical Medicine, University of Gothenburg, Sweden; ^11^Department of Human Genetics, David Geffen School of Medicine at University of California Los Angeles (UCLA), Los Angeles, CA, USA; ^12^Institute for Precision Health, School of Medicine, UCLA, Los Angeles, CA, USA; ^13^Research Program for Clinical and Molecular Metabolism, Faculty of Medicine, University of Helsinki, Helsinki, Finland; ^14^Research Unit for Biomedicine and Internal Medicine, Faculty of Medicine, University of Oulu, Oulu, Finland; ^15^Medical Research Center Oulu, Oulu University Hospital and University of Oulu Finland; ^16^Biocenter Oulu, University of Oulu, Oulu, Finland; ^17^Turku PET Centre, University of Turku, Turku, Finland; ^18^Obesity Research Unit, Research Program for Clinical and Molecular Metabolism, Faculty of Medicine, University of Helsinki, Helsinki, Finland; ^19^Obesity Center, Endocrinology, Abdominal Center, Helsinki University Central Hospital and University of Helsinki, Helsinki, Finland; ^20^Department of Medicine, Endocrinology and Clinical Nutrition, Kuopio University Hospital, Kuopio, Finland

**Supplementary methods 1**

The XFe96 Sensor Cartridge was hydrated with XF Calibrant solution overnight (pH-7.4, 37°C, non-CO2 atmosphere). On the day of assay, cells were incubated with XF Assay Medium (including glutamine) supplemented with 17.5mM D-glucose and 0.5mM of sodium pyruvate (pH-7.4) for 1 hour in a non-CO2 incubator at 37°C. For all the experiments with Seahorse Mito Stress Kit, oligomycin (5µM), Carbonyl cyanide-4 (trifluoromethoxy) phenylhydrazone (FCCP) (5µM), and a mixture of rotenone/antimycin A (2µM) were injected sequentially after baseline measurements. OCR and ECAR values are presented without an assay inhibitor challenge (baseline readings), and in the presence of an assay inhibitor challenge, the metabolic potential (%) is calculated by dividing the stressed (post-injection) values by the baseline values (pre-injection) × 100. Data were expressed as pmol of O2 per minute and normalized by total DNA content measured by the Cyquant Cell proliferation kit (Thermo Fisher Scientific, Carlsbad, CA) following the manufacturer’s instruction.

**Supplementary methods 2**

DNA concentrations were measured using a Qubit4 Fluorometer (Thermo Scientific). A standard curve with 8, 2, 0.5, 0.1, and 0.02 ng of DNA was prepared from the pooled samples. mtDNA was quantified using primers for genomic regions mitochondrially encoded 16S ribosomal RNA (16S), cytochrome B (CYTB) and D-loop (D-loop). For ncDNA we used amplified regions of amyloid beta precursor protein (APP), hemoglobin subunit beta (HBB), and beta-2-microglobulin (B2M). Sensifast SYBR Lo‐ROX Kit (Bioline, BIO 94050) with QuantStudio 6 pro-Real-Time PCR System (Thermo Fisher, Landsmeer, The Netherland) with the standard curve method in Excel was used to calculate this assay.

**Supplementary methods 3**

Mitochondrial morphology was analyzed using acquired image stacks processed with ImageJ (v1.54d) software. The images were separated into individual channels and only the channel containing mitochondrial staining was retained. Deconvolution was performed using the Richardson-Lucy total variation algorithm with a wavelength of 0.0001 and 10 iterations via the Deconvolution Lab2 (v2.1.2) tool (Sage et al., 2017). The point spread function (PSF) model utilized for deconvolution was a theoretical PSF generated using the Richards & Wolf 3D optical model at a wavelength of 598 nm via the PSF generator (v18.12.2017) plugin. The images were subsequently converted to 8-bit and pixel/voxel units were set to microns. Thresholding was performed on each image, followed by mitochondrial morphological and networking analysis of 3D stacks using the Mitochondria Analyzer (v2.3.1) plugin with default parameters.

**Supplementary Figure 1**


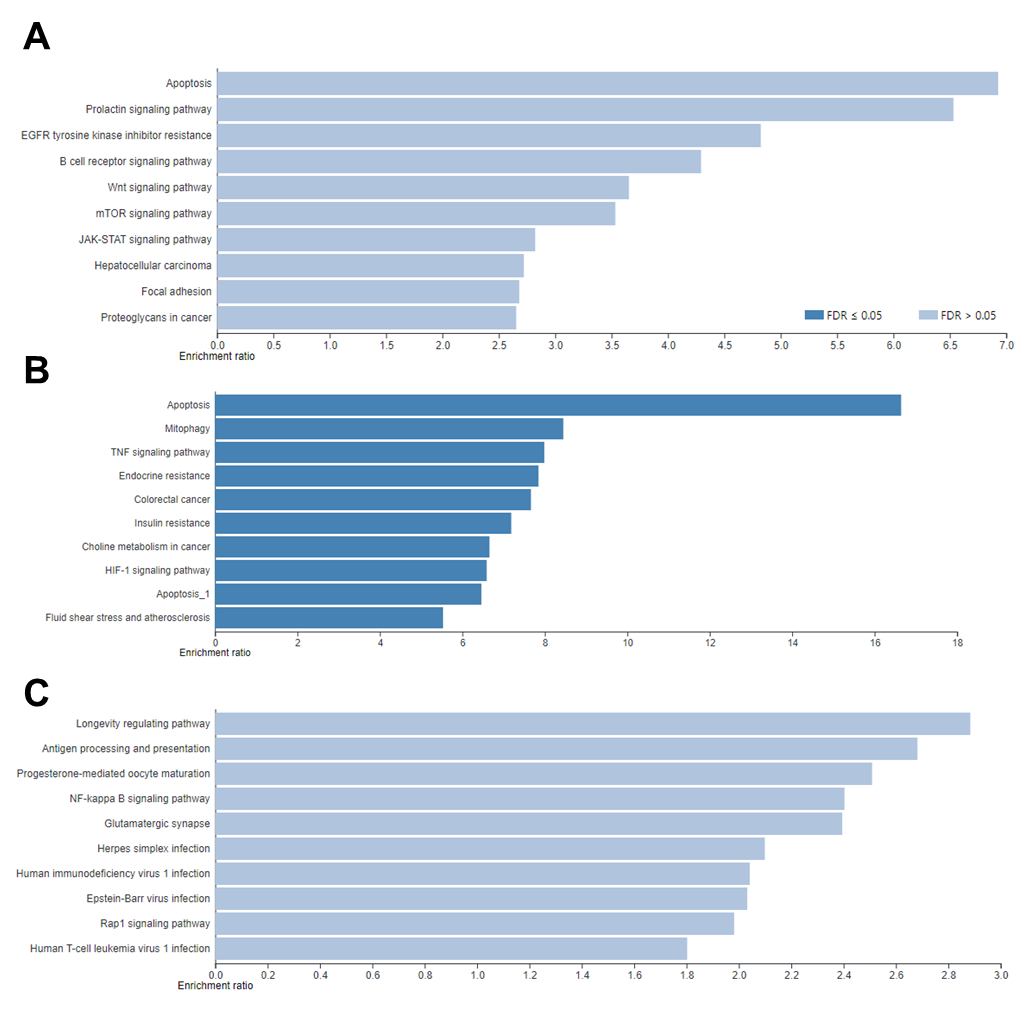


**Supplementary Figure 1:** Enriched pathways from RNA sequencing of global, mitochondrial transcriptomics, and annotated genes of DNA methylation in the human liver corresponding to the significantly associated genes with serum IPA signal intensities in KOBS without T2D subjects. **A-C)** Apoptosis and longevity were the top canonical pathways based on a total of 268 genes from global liver transcriptomics, 119 genes from liver mitochondrial genes, and DNA methylation genes that map to 3092 CpGs sites that were nominally associated with IPA (Webgestalt analysis).

**Supplementary Figure 2**


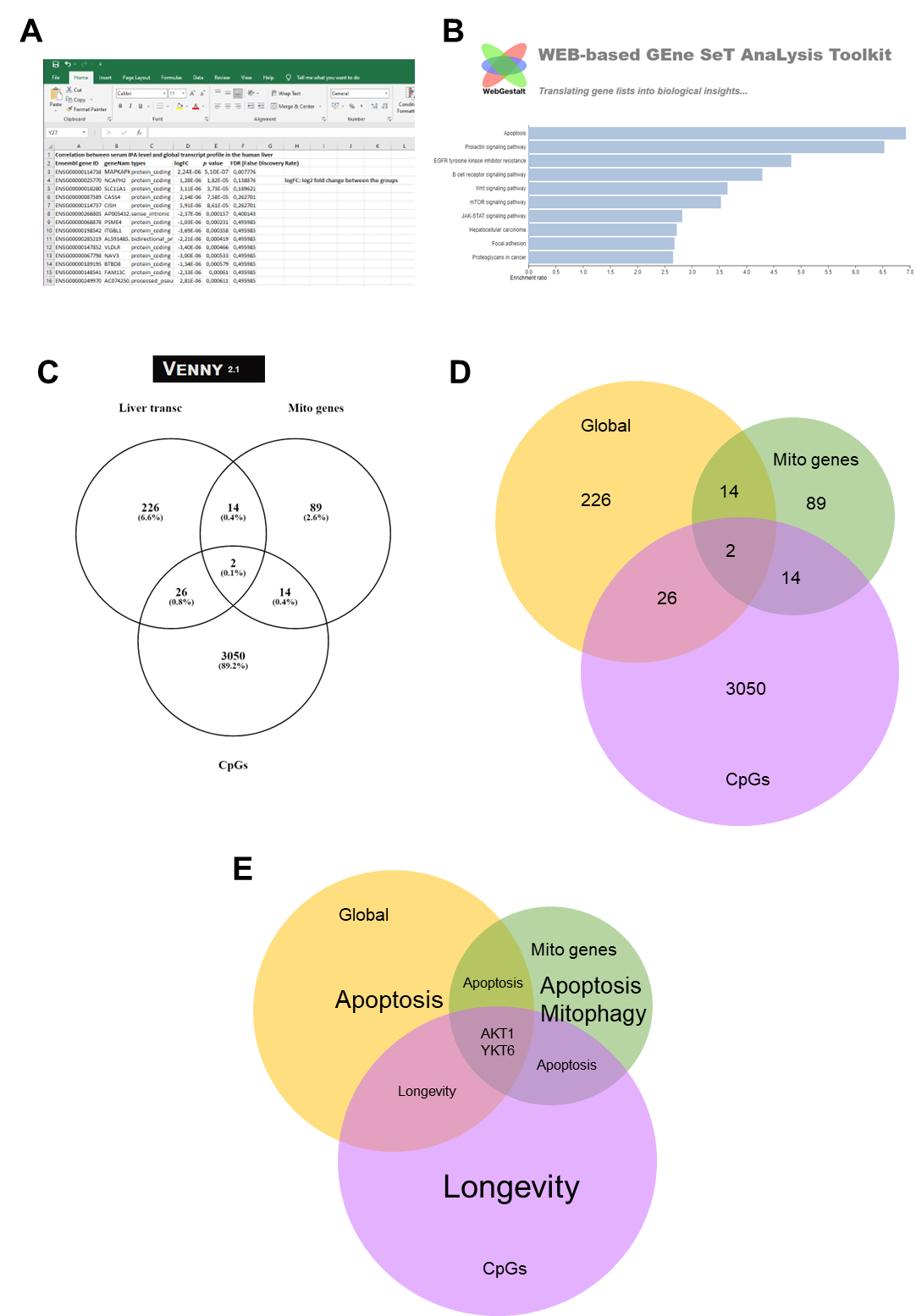


**Supplementary Figure 2: Schematic view of the step by step overlapping genes in the liver transcriptomics pathway analysis. A-B)** Pathway analysis was done separately for 268 transcripts (nominal p<0.01), 119 mitochondrial-related transcripts (nominal p<0.05), and 4350 CpGs mapped to 3093 transcripts in the liver all of which were associated with circulating serum IPA levels using WEB-based Gene SeT Analysis Toolkit (WebGestalt). **C)** The freely available Venny DB (version 2.1.0) tool was used to find the overlapping genes. **D-E)** The Venn diagram representing 268 global transcripts, 119 mitochondrial-related transcripts, and DNA methylation of transcripts that map to 3092 CpGs sites correlated with serum IPA levels (p-value<0.01 for global and DNA methylation, p-value<0.05 for mitochondrial-related transcripts) and the pathway analysis from that overlapping. The main overlapped transcripts are shown in the middle (*AKT1* and *YKT6*).

**Supplementary Figure 3**


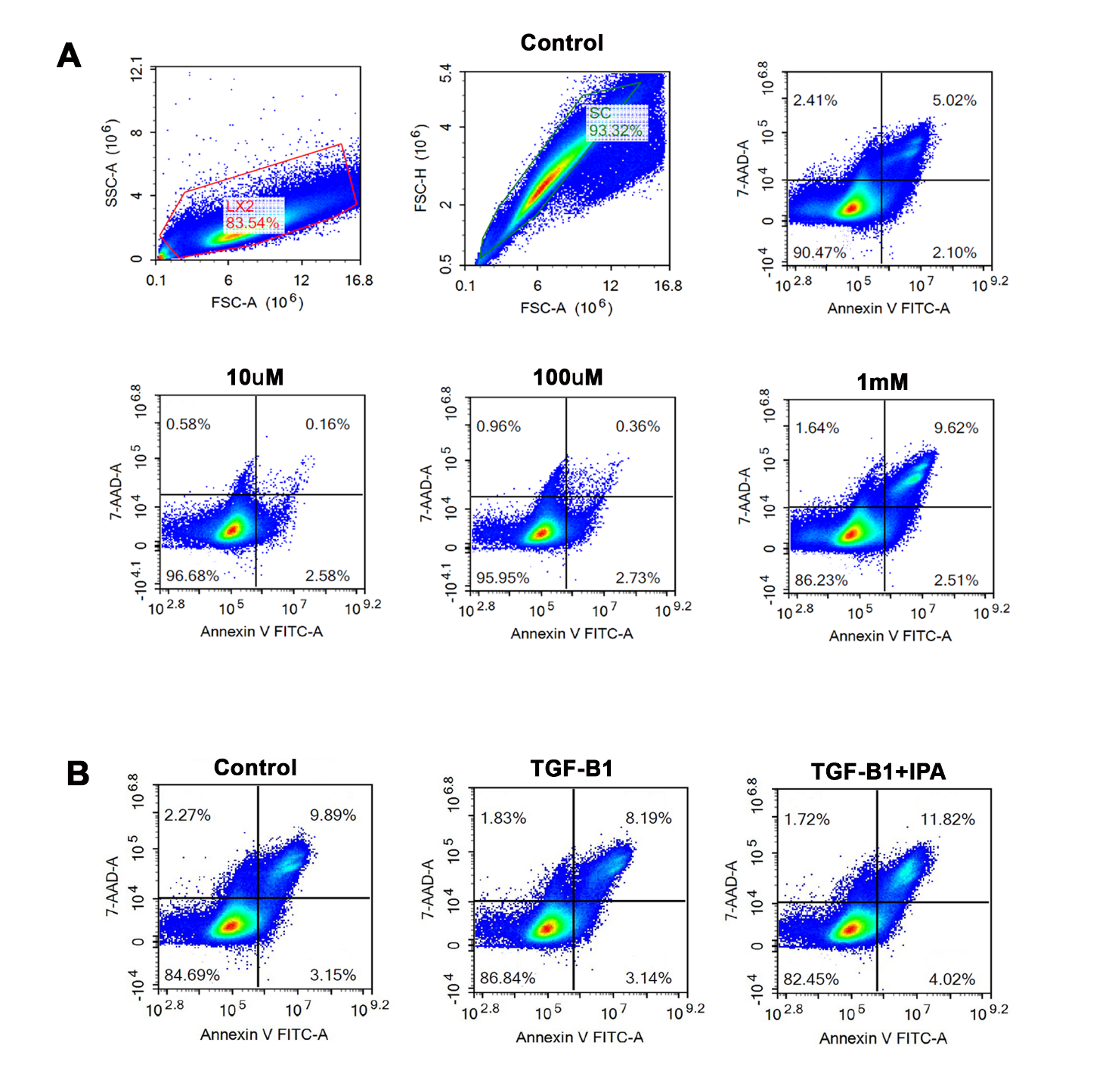


**Supplementary Figure 3: IPA induces apoptosis in LX-2 cells**. **A-B)** Representative images from flow cytometry analysis. The analysis was performed using a double staining method with Annexin V and 7-AAD. **A)** Cells were incubated with 10 µM, 100 µM, and 1 mM of IPA for 24 hours and gated into lower right and upper right quadrants, which represented early (Annexin V ^+^/ 7AAD^-^) and late apoptotic (Annexin V ^+^/ 7AAD^+^). Lower left quadrants mean viable cells (Annexin V ^-^/ 7AAD^-^) and upper left quadrants mean necrotic cells (Annexin V ^-^/ 7AAD^+^). **B)** Cells were incubated with TGF-β1 (5ng/ml) and 1 mM of IPA for 24 hours in non-serum media and gated into lower right and upper right quadrants, which represented early (Annexin V ^+^/ 7AAD^-^) and late apoptotic (Annexin V ^+^/ 7AAD^+^). Lower left quadrants mean viable cells (Annexin V ^-^/ 7AAD^-^) and upper left quadrants mean necrotic cells (Annexin V ^-^/ 7AAD^+^).

**Supplementary Figure 4**


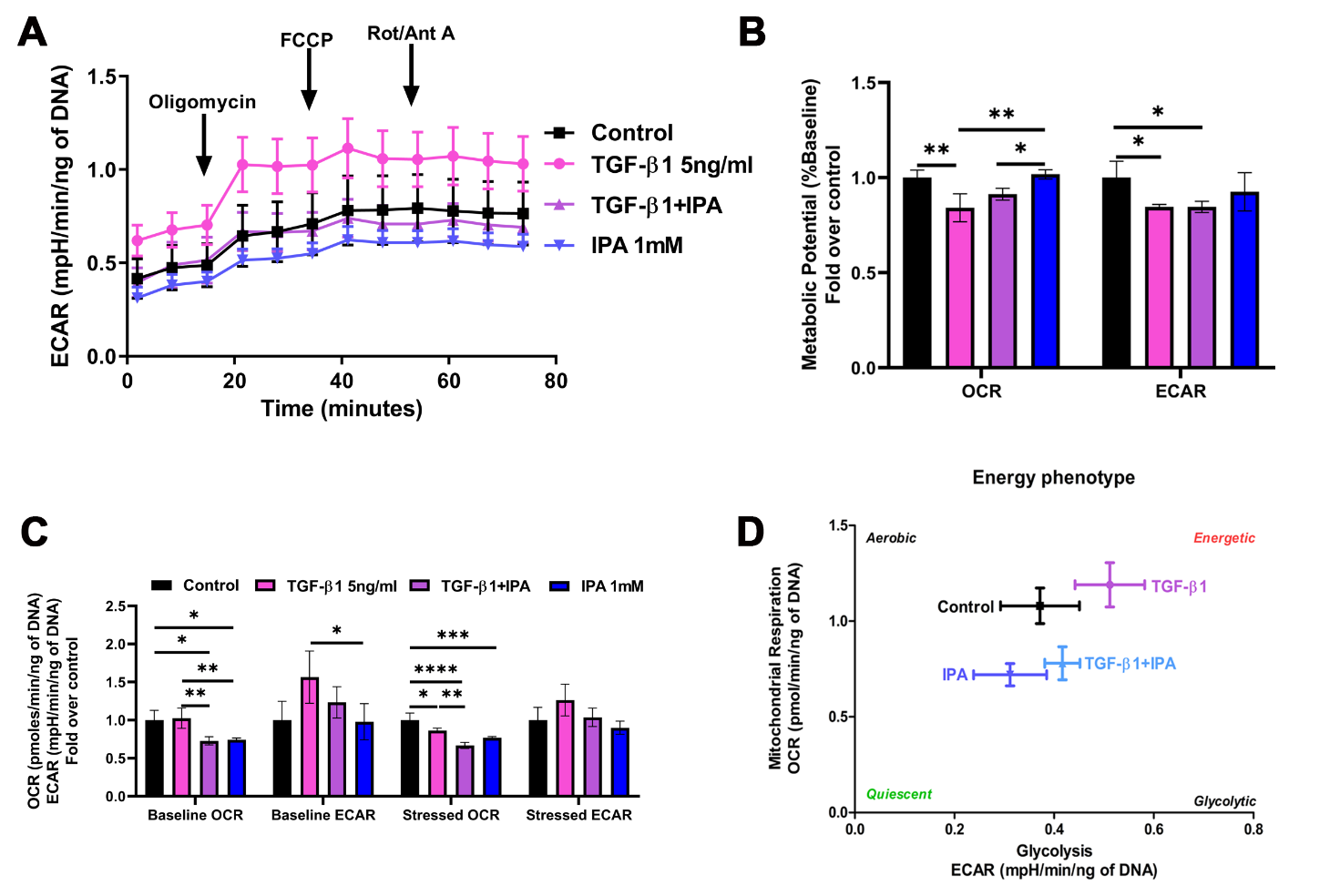


**Supplementary Figure 4: IPA can induce a less energetic profile in LX-2 cells.** Results from the Seahorse XF Energy Phenotype Test Report Generator shown for OCR and ECAR. Cells were incubated with TGF-β1 (5ng/ml) and 1 mM of IPA for 24 hours. **A)** ECAR respiration curves. **B, C**) metabolic potential ((stressed OCR or ECAR/baseline OCR or ECAR) × 100%)), and **D**) energy phenotype graph. All the measurements were normalized for the DNA amount by CyQuant kit. OCR: Oxygen consumption rate, ECAR: Extracellular acidification rate. Data were shown as mean ± SD, n= 5 independent experiments. One-way ANOVA with Bonferroni´s *post hoc* test was used for statistical comparisons. *p<0.05 **p <0.01***p<0.001 and ****p<0.0001.

**Supplementary Figure 5**


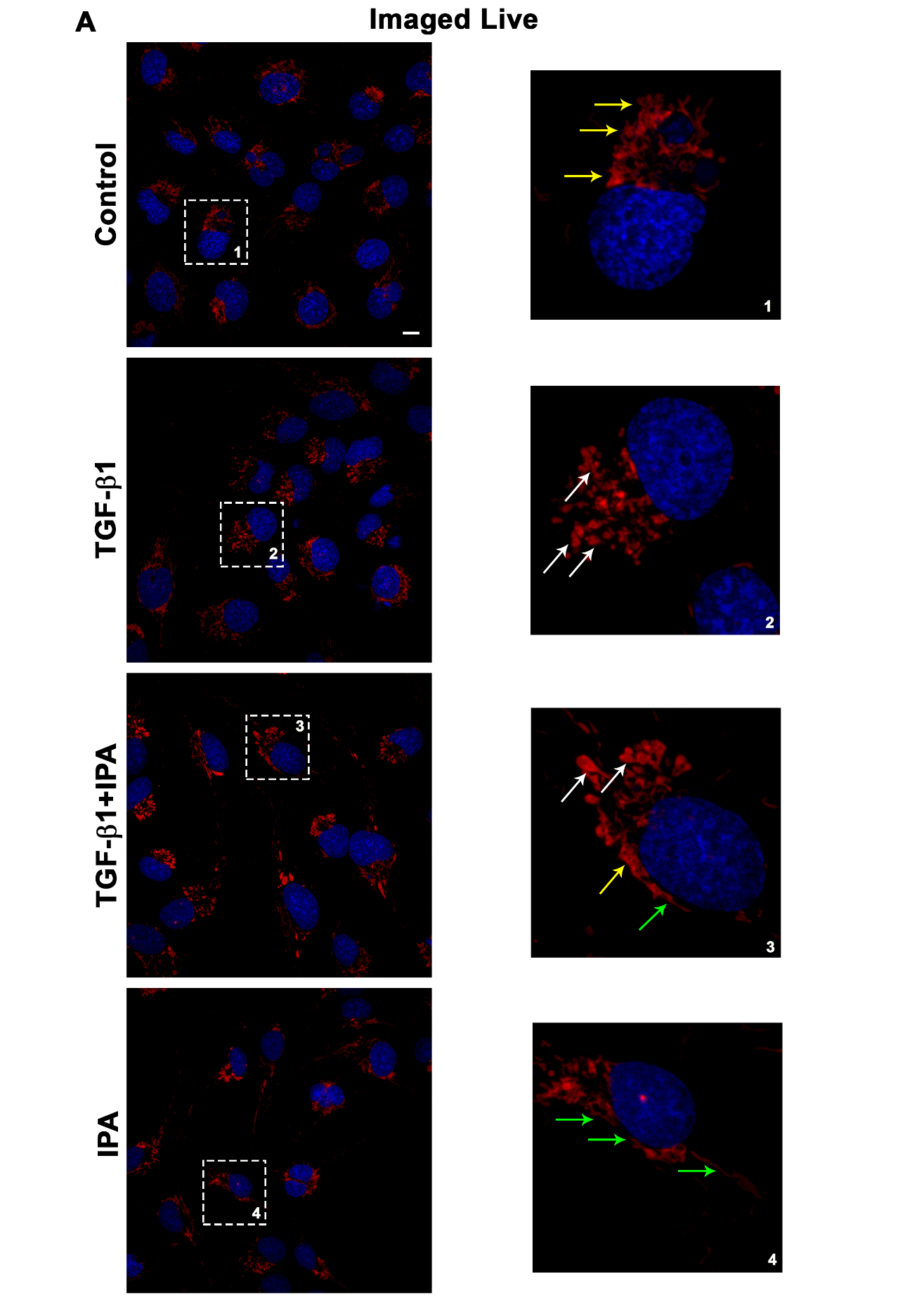


**Supplementary Figure 5:** IPA alters mitochondrial dynamics in LX-2 cells. A) Representative confocal images of living LX-2 cells with TGF-β1 (5ng/ml) and IPA 1mM in non-serum media for 24 hours showing the mitochondrial networking stained with Mitotracker® Red CMXRos and blue nucleus with DAPI. We acquired 10 Z-stack images from each sample type. Each Z-stack included 30 slices, with each slide having a thickness of 9.86 µm. Arrows in 1,2,3 and 4 square x3 zoom indicate white round spheres shape mitochondria, green arrow filamentous and elongated mitochondria, and yellow arrow intermediate mitochondria. All data were presented with at least 15 images per group, n= 3 independent experiments. Scale bar, 10 µm.

**Supplementary Figure 6**


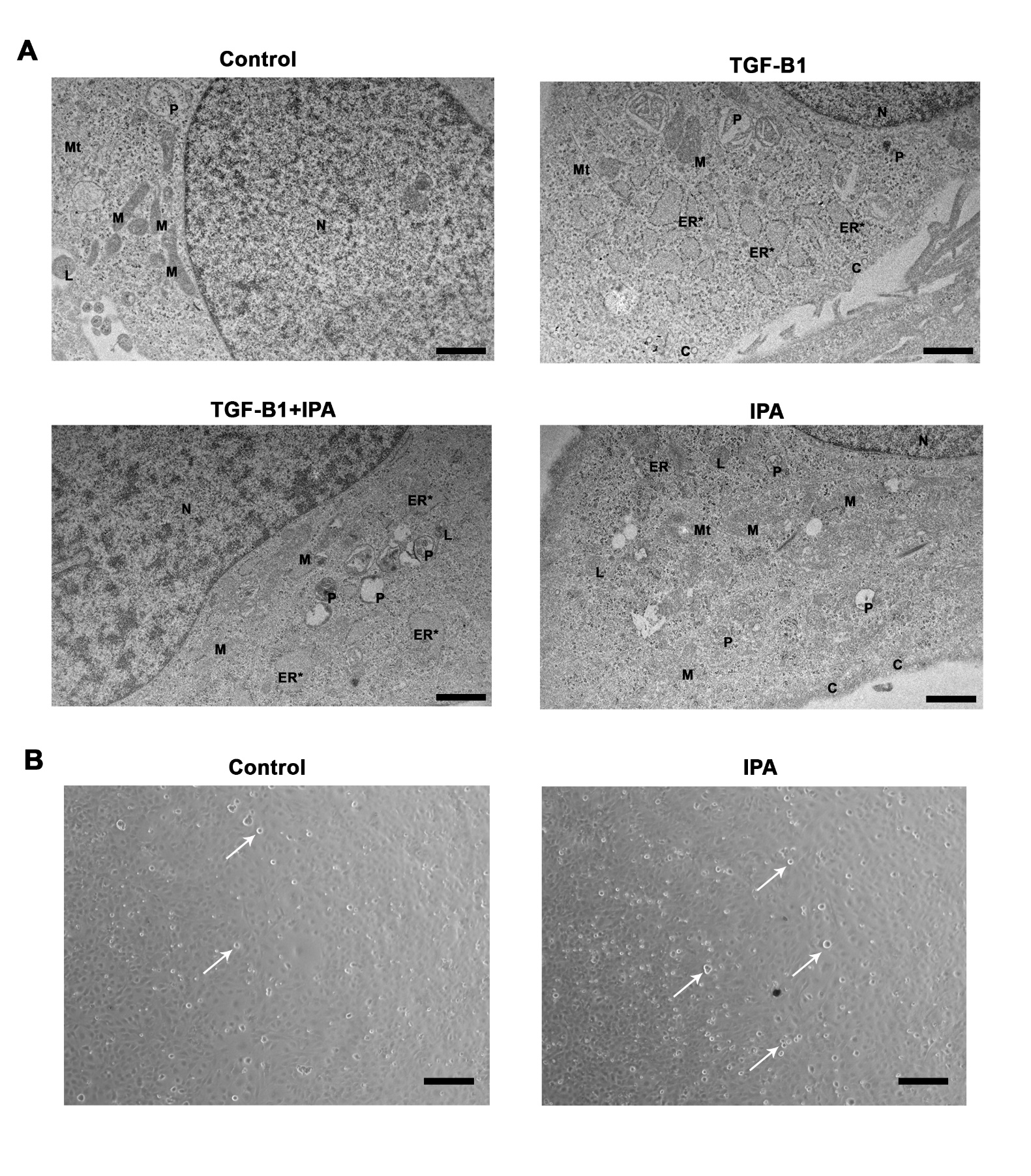


**Supplementary Figure 6: Impact of IPA on cell morphology in LX-2 cells**.**A)**Representative images from ultrastructural analysis assessed by TEM on LX-2 cells showing: Nucleus (N), mitochondria (M), lysosome (L), phagolysosome (P), rough endoplasmic reticulum (ER), dilated ER (ER*), caveolae (C), microtubules (Mt). Cells were incubated with TGF-β1 (5ng/ml) and 1 mM of IPA for 24 hours in non-serum media. Scale bar 1µm. **B)** Images from phase contrast microscopy 24 hours after IPA treatment showing the apoptotic cells (white arrows). All data were presented with at least 15 images per group, n=3 independent experiments. Magnification 50x.

**Supplementary Figure 7**

**
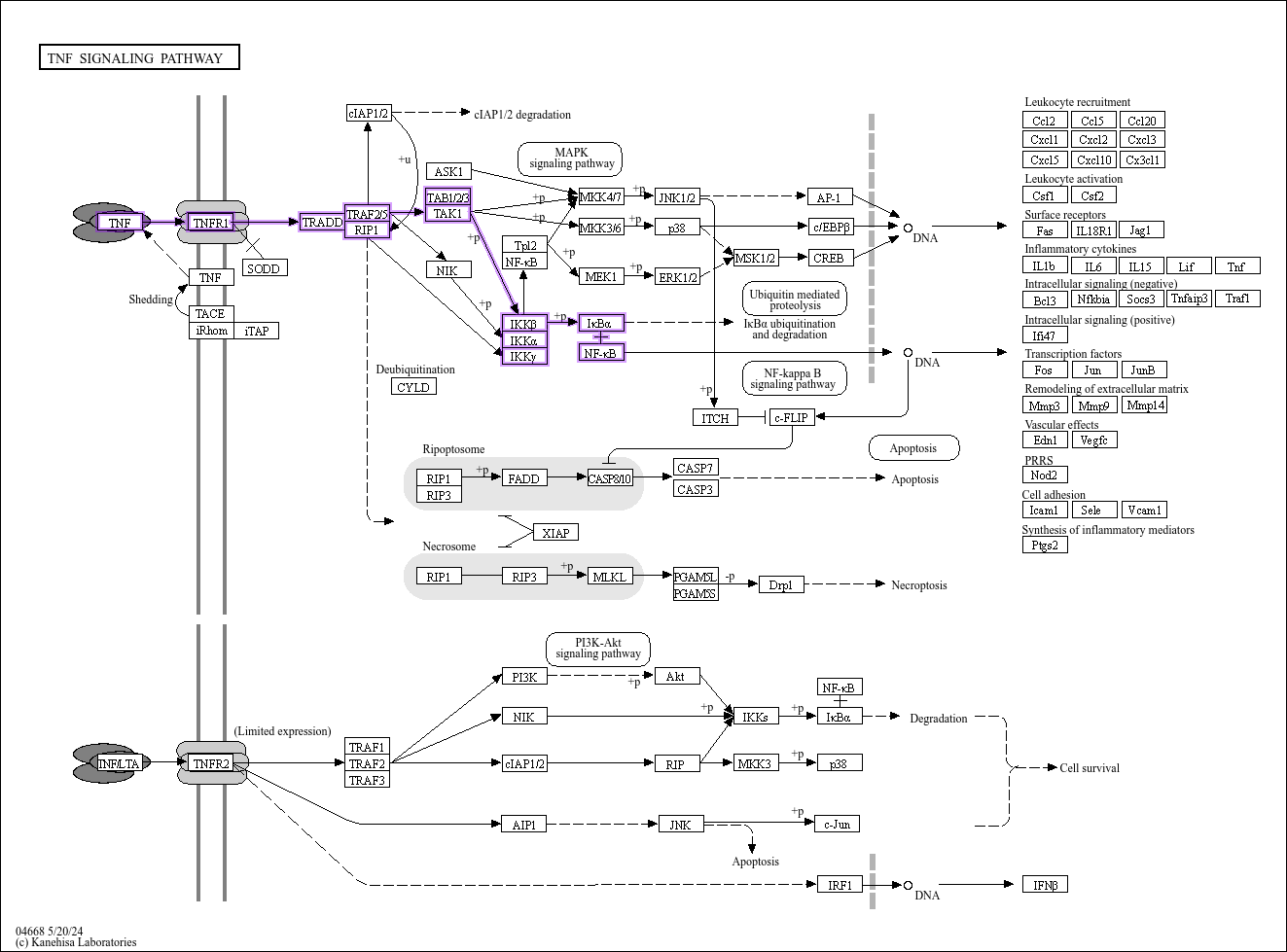
**

**Supplementary Figure 7: KEGG pathway analysis of the TNF signaling pathway that can induce IPA pro-survival or proapoptotic signals on HSCs.** The link between the genes commonly regulated in TNF-NFκB signaling pathway datasets was highlighted using purple color. Modified from kegg.jp/pathway/map04668.

**Table S1**: Clinical characteristics and liver histology of study participants according to histological liver phenotype.

|  | Normal liver | Simple steatosis | NASH | p ^a^ |
| --- | --- | --- | --- | --- |
| Total, N (male/female) | 51 (12/39) | 22 (6/16) | 9 (4/5) | n.s. |
| Age (years) | 45.8±9.4 | 47.4±8.7 | 47.5±10.5 | n.s. |
| BMI (kg/m^2^) | 42.2±5.0 | 43.4±4.8 | 44.9±6.0 | n.s. |
| fS-Total cholesterol (mmol/l) | 4.2±0.7 | 4.5±0.9 | 4.3±1.1 | n.s. |
| fS-HDL cholesterol (mmol/l) | 1.1±0.3 | 1.0±0.2 | 1.0±0.2 | n.s. |
| fS-LDL cholesterol (mmol/l) | 2.4±0.6 | 2.7±0.8 | 2.5±0.8 | n.s. |
| fS-Triglycerides (mmol/l) | 1.2 (0.9–1.9) | 1.3 (0.9–2.2) | 1.7 (1.1–2.7) | n.s. |
| fP-glucose (mmol/l) | 5.6±0.6 | 5.9±1.0 | 6.3±0.7* | 0.021 |
| fS-insulin (mU/l) | 13.8 (9.1–18.2) | 15 (11.–22.6) | 21.5 (14.9–30.3)* | 0.033 |
| Steatosis grade, N |  |  |  |  |
| <5% | 51 | 0 | 0 |  |
| 5-33% | 0 | 21 | 4 |  |
| 33-66% | 0 | 1 | 3 |  |
| >66% | 0 | 0 | 2 |  |
| Lobular inflammation, N | 0 | 0 | 9 |  |
| Ballooning, N | 0 | 0 | 8 |  |
| Fibrosis, N (stage range) | 0 | 0 | 9 (1-2) |  |

One-way ANOVA continuous variable or χ2 test. Data are present as mean + SD ^a^ p<0.05 after ANOVA; *p<0.05 when compared to normal liver after Bonferroni *post hoc* correction. N- number of individuals, BMI- Body mass index, fS- fasting serum, fP- fasting plasma, HDL- high-density lipoprotein, LDL- low-density lipoprotein.

**Table S2:** Primers Sequences for real-time q-PCR analyses of different genes using SYBR Lo‐ROX Kit

|  | Forward | Reverse | Genbank reference | Amplicon |
| --- | --- | --- | --- | --- |
| *α‐SMA* | 5′‐AATCCTGACCCTGAAGTACC | 5′‐TAGAAAGAGTGGTGCCAGAT | NM_001613.4 | 84 bp |
| *COL1A2* | 5′‐AGCTGTTGGTAACGCTGGTC | 5′‐CAGGATTACCAGGAGGTCCA | NM_000089.4 | 98 bp |
| *MMP2* | 5′‐AAGTATGGCTTCTGCCCTGA | 5′‐ATTTGTTGCCCAGGAAAGTG | NM_001613.4 | 97 bp |
| *TIMP1* | 5′-CAAGATGTATAAAGGGTTCCAAGC | 5′-TCCATCCTGCAGTTTTCCAG | NM_003254.3 | 148 bp |
| *DRP1* | 5´-GATGCCATAGTTGAAGTGGTGAC | 5´-CCACAAGCATCAGCAAAGTCTGG | NM_012062 | 134 bp |
| *MFN1* | 5´-GGTGAATGAGCGGCTTTCCAAG | 5´-TCCTCCACCAAGAAATGCAGGC | NM_033540.3 | 135 bp |
| *OPA1* | 5´-GTGGTTGGAGATCAGAGTGCTG | 5´-GAGGACCTTCACTCAGAGTCAC | NM_130835.3 | 130 bp |
| *FIS1* | 5´-CAAGGAACTGGAGCGGCTCATT | 5´-GGACACAGCAAGTCCGATGAGT | NM_016068.3 | 124 bp |
| *BCL-2* | 5´-GTGCCTGCTTTTAGGAGACCGA | 5´-GAGACCACACTGCCCTGTTGATC | NM_000633.3 | 128 bp |
| *BAX* | 5´-TTTGCTTCAGGGTTTCATCCA | 5´-GAGACACTCGCTCAGCTTCTTG | NM_138764.5 | 115 bp |
| *Casp-3* | 5´-GTAGAAGTCTAACTGGAAAACCCAA | 5´-CATGTCATCATCAACACCACTGTCT | NM_004346.4 | 104 bp |
| *Casp-8* | 5´-GAGTCATCTCTGTTCTGCTTTAGGA | 5´-GTGAAAACACTTCCCTCCAGC | NM_001400654.1 | 118 bp |
| *BIK* | 5´-CTTGATGGAGACCCTCCTGTATG | 5´-AGGGTCCAGGTCCTCTTCAGA | NM_001197.5 | 91 bp |
| *APAF-1* | 5´-TGGCAGTGGTTGCTTTGTCCCAGT | 5´-GGAGAAAACATCACACCATGAACC CAACTT | NM_181861.2 | 115 bp |
| *RPLOP0* | 5´-GGCGACCTGGAAGTCCAACT | 5´-CCATCAGCACCACAGCCTTC | NM_053275.4 | 149 bp |
| *PPIA* | 5´-ACGTGGTATAAAAGGGGCGG | 5´-TGCCATCCAACCACTCAGTC | NM_021130.3 | 98 bp |

**Table S3:** Primers Sequences for RT-PCR analyses using TaqMan™ Fast Advanced Master Mix

| **Gene symbol** | **Assay ID** |
| --- | --- |
| **IKBKB** | **Hs00233284_m1** |
| **NFKBIA** | **Hs00153283_m1** |
| **NFKBIB** | **Hs00182115_m1** |

**Table S4:** Primers Sequences for RT-PCR analyses for mtDNA and ncDNA

| *mtDNA and ncDNA* | Forward | Reverse |
| --- | --- | --- |
| *16S* | 5´-GGGGCGACCTCGGAGCAGAA | 5´-ATAGCGGCTGCACCATCGGGA |
| *CYTB* | 5´-GCCTGCCTGATCCTCCAAAT | 5´-AAGGTAGCGGATGATTCAGCC |
| *DLOOP* | 5´-CATCTGGTTCCTACTTCAGGG | 5´-CCGTGAGTGGTTAATAGGGTG |
| *APP* | 5´-TGTGTGCTCTCCCAGGTCTA | 5´-CAGTTCTGGATGGTCACTGG |
| *B2M* | 5´-TGCTGTCTCCATGTTTGATGTATCT | 5´-TCTCTGCTCCCCACCTCTAAGT |
| *HBB* | 5´-CAGGTACGGCTGTCATCAGTTAG | 5´-CATGGTGTCTGTTTGAGGTTGCT |
